# Adaptation of a Danish online version of the Oxford Physical Activity Questionnaire (OPAQ) for secondary school students—a pilot study

**DOI:** 10.1186/s40814-022-01108-x

**Published:** 2022-07-25

**Authors:** Charlotte Raadkjær Lykkegaard, Sonja Wehberg, Frans Boch Waldorff, Jens Søndergaard, Sinead Holden

**Affiliations:** 1 The Research Unit of General Practice, Department of Public Health, University of Southern Denmark, Odense, Denmark; 2 Section of General Practice and The Research Unit for General Practice, Department of Public Health, University of Copenhagen, Copenhagen, Denmark; 3 Centre for General Practice at Aalborg University, Aalborg University, Aalborg, Denmark; 4 Department of Health Science and Technology, Aalborg University, Aalborg, Denmark; 5 UCD Clinical Research Centre, School of Medicine, University College Dublin, Dublin, Ireland

**Keywords:** Adolescent, Physical activity, PROM, OPAQ

## Abstract

**Objective:**

To adapt and partly validate a Danish online version of the patient-reported outcome measure (PROM) Oxford Physical Activity Questionnaire (“OPAQ”) and evaluate mobile phones and tablets as data capturing tool to identify potential problems and deficiencies in the PROM prior to implementation in the full study.

**Methods:**

The OPAQ was translated into Danish by a formalised forward-backward translation procedure. Face validity was examined by interviewing 12 school students aged 10–15, recruited from two Danish public schools. After modifications, the online version of the Danish OPAQ was pilot tested in a convenience sample of seven school students for 1 week. Simultaneous objective accelerometer data were captured during the registration period.

**Results:**

No major challenges were identified when translating OPAQ. Based on the interviews, the Danish version of OPAQ was perceived to be easy to understand in general, and the questions were relevant for tracking activities during the week. Five of the 12 participants had difficulties with understanding the introductory question: “what is your cultural background” in the original OPAQ. The interviews revealed that the participants recalling 7 days forgot to record some of the physical activity they had done during the week, indicating issues with the weekly recall method.

After transforming to the online version, this was reported to be easy and quick to fill in (taking 1–3 min per day), and participants reported the daily design was helpful to remember activities. There was good correspondence between the online version and objective actigraphs with a tendency to underreport. Six participants reported 10–60 min less moderate to vigorous physical activity compared to the actigraphs, while one participant reported 3 min more.

**Conclusion:**

Participants found the online OPAQ quick and easy to complete during a 1-week period. Completing daily rather than weekly may help limit issues with recall. Overall, there was good agreement between the objective actigraphs and the OPAQ, though the OPAQ tended to slightly underreport moderate to vigorous physical activity.

The Danish online version of OPAQ may be useful for capturing school students’ physical activity when objective measures are not feasible.

**Supplementary Information:**

The online version contains supplementary material available at 10.1186/s40814-022-01108-x.

## Key messages

1. What uncertainties existed regarding the feasibility?
  Is the Danish online version of the Oxford Physical Activity Questionnaire feasible for secondary school students to respond to daily use of mobile devices?
  Is the Danish online version of the Oxford Physical Activity Questionnaire more feasible than the weekly paper report due to recall?
  Is the Danish online version of the Oxford Physical Activity Questionnaire useful to capture physical activity in school students when objective measures are not feasible?
2. What are the key feasibility findings?
  The online version of OPAQ was found easy and quick to complete by the students, and the daily design was helpful regarding the recall of physical activity.
  Using a smartphone application was a success.
  The online version of OPAQ is useful for capturing school students’ physical activity when objective measures are not feasible keeping in mind that the OPAQ tend to underreport the students’ daily moderate to vigorous physical activity level.
3. What are the implications of the feasibility findings for the design of the main study?
  Due to the small sample in this study, a full validation of the online version needs to be conducted before progressing to our large-scale cohort study.

## Introduction

The Oxford Physical Activity Questionnaire (OPAQ) is a questionnaire designed to assess the physical activity patterns of secondary school students [1].

Physical activity (PA) is an important component of public health agendas [2] and an integral factor for facilitating optimal bone growth and muscle development, mental health, personal development, and academic learning [2–6]. The World Health Organization (WHO) defines PA as “any bodily movement produced by skeletal muscles that requires energy expenditure” [7]. PA in childhood is associated with a higher level of PA later in life and reduced risk of developing obesity and chronic diseases [3, 8, 9]. Higher intensity activities or longer durations are essential [10]. For adolescents aged 10–17 years old, WHO recommends at least 60 min of moderate- to vigorous-intensity PA daily [9]. Many children and adolescents fail to meet these minimum activity recommendations [11–13].

This is a pilot study for a future population-based cohort investigating physical activity and musculoskeletal injuries in Danish school students aged 10–15.

Objective measures of PA using accelerometers such as actigraphs represent the gold standard but are expensive and time-consuming for both data capturing and analysis. This makes it difficult to implement in large cohorts, as well as in “the real world”. Therefore, a validated and reliable self-reported measure tool is needed for this population-based cohort study. Questionnaires for children and adolescents need to be designed specifically due to differences in cognition and vocabulary compared to adults. This is critical to prevent misunderstanding and avoiding potential difficulties in recalling activity patterns for this age group.

The self-administered questionnaire Oxford Physical Activity Questionnaire (OPAQ) is designed specifically for secondary school students [1, 14] (Additional file 1). The questionnaire is meant to be completed retrospectively and requires participants to report the amount of time spent in different physical activities during the past 7 days. It takes approximately 15 min in total to be completed by the respondents [1].

The primary aim of this study was to adapt and partly validate a Danish online version of the patient-reported outcome measure (PROM) “OPAQ” and evaluate mobile phones and tablets as data capturing tool to identify potential problems and deficiencies in the PROM prior to implementation in the full study.

The objectives were to (1) translate and cross-culturally adapt the original OPAQ from English into Danish and investigate face validity by cognitive interviews in Danish secondary school students, and (2) to transfer the Danish version into an online version and test if it is feasible for secondary school students to respond via their mobile phones and tablets (2a) and compare their results to objectively measured physical activity by accelerometer (2b).

## Material and methods

### Participants

Inclusion criteria for all steps of this study were that the participants should be aged 10–15, be able to read and understand Danish fluently, attend a Danish public school, and be between 4th and 9th grade (inclusive). In addition, to participate in piloting the online version of the Danish OPAQ, the participants needed to have access to a smartphone.

### Ethics

The Ethical Committee of the Region of Southern Denmark (Project ID: S-20200054) waived the need for ethical approval as the study only pertained nonsensitive self-reported questionnaires. All participants and their parents provided informed consent prior to participation in the study.

### Oxford Physical Activity Questionnaire

**Fig. 1:**
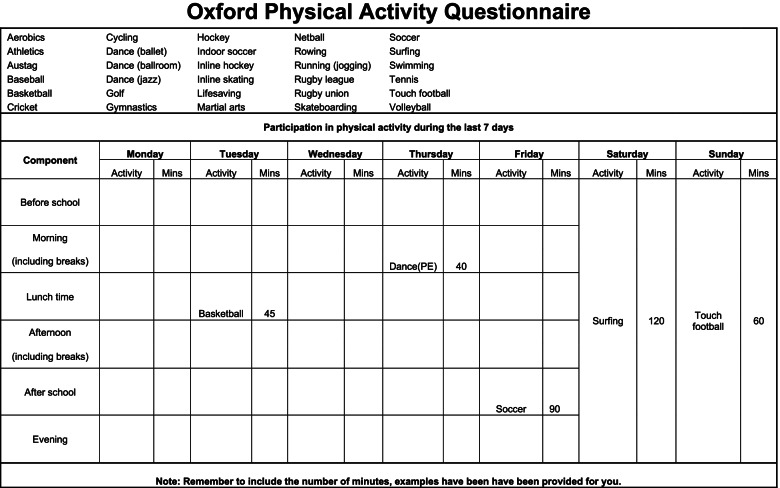
The week timetable of OPAQ

The OPAQ was developed in 2008 by David R. Lubans (University of Newcastle, Australia), Kathy Sylva (University of Oxford, UK), and Zane Osborn (University of Newcastle, Australia) [1]. OPAQ is one of five questionnaires recommended by a systematic review “Physical Activity Questionnaires for Youth” [14] and the only one of them capturing data on both free time PA and organised sports during and after school. The original questionnaire was validated by comparing the self-reported data with Caltrac accelerometer counts. Correlations were related to vigorous physical activity (*r* = 0.33, *p* = 0.01) and moderate to vigorous activity (*r* = 0.32, *p* = 0.02) [1]. The questionnaire consists of seven demographic questions and a week timetable format (similar to ones students often use for their weekly schedule (Fig. 1)), to record the amount of time spent on any moderate to vigorous physical activity the respondent has done the past 7 days in relation to frequency (days) and duration (hours or minutes) of the activity [1].

### Linguistic translation and cross-cultural adaptation of OPAQ

Before translation, permission was obtained to perform a Danish translation and cross-cultural adaptation from the authors of the original OPAQ [1]. A formalised 5-step translation procedure was performed as suggested by Beaton et al. [15]:

1. First, the OPAQ was translated independently by three separate individuals with and without domain knowledge. Specifically, this included a participant with a Master of Science in Sports and Health, a master in physiotherapy, and a naive translator without any medical or physical activity background.
2. The three Danish translations were discussed between the three translators and one of the researchers; any discrepancies were reconciled, and a final translation was agreed upon in the group.
3. The Danish version was then back translated by two independent native English speakers living in Denmark-speaking Danish. Both translators have moved to Denmark as adults and have lived in Denmark for 19 years. They have both passed the Danish national language test for professional proficiency which is equivalent to level C1 on the Common European Framework of Reference for Languages [16]. The two translators were not familiar with the original OPAQ and were not medically trained. These two backtranslations were then sent to the authors of the original OPAQ for approval.
4. After approval, the “expert committee” consisting of all 5 translators discussed a few minor disagreements with wordings and sentence structures and agreed on a final version.
5. The final version of the Danish questionnaire was evaluated to assess whether the original idea is maintained and if the translated questionnaire is easy to understand by the school students [17, 18].

### Face validity of the translated Danish version

A convenience sampling composed of 12 school students aged 10–15 was recruited from two Danish public schools. Of these, seven completed the paper questionnaire at the end of the week (as per the original), while five received the questionnaire in the beginning of the week and completed it every evening.

Students completed a short interview afterwards with one of the researchers. They were interviewed in their private homes. The interview was semi-structured following an interview guide. The aim was to investigate any problems with the questionnaire including wordings, comprehension, time use, ease of use, and success of recalling activities. The students were also encouraged to give suggestions for improvements where they considered it appropriate. During the interview, notes were taken by the researcher and were used to improve the online version.

### Evaluation of the online version

The online version was designed to be completed at the end of each day to limit recall bias, meet the school students desired use of mobile devices, and to increase accuracy and efficiency of data collection. The online Danish version was captured using the MyCap application on the students’ smartphones or tablets. MyCap is a mobile application which is synchronised to REDCap (Research Electronic Data Capture) hosted at OPEN, Odense University Hospital, Denmark. REDCap is a secure, web-based software platform designed to support data capture for research studies [19, 20].

Finally, to ease the use of OPAQ, the seven demographic questions from the original version were included in a baseline questionnaire, which the students and their parents needed to complete when signing up for the study. For details on the baseline questionnaire, please refer to Additional file 2.

**Fig. 2:**
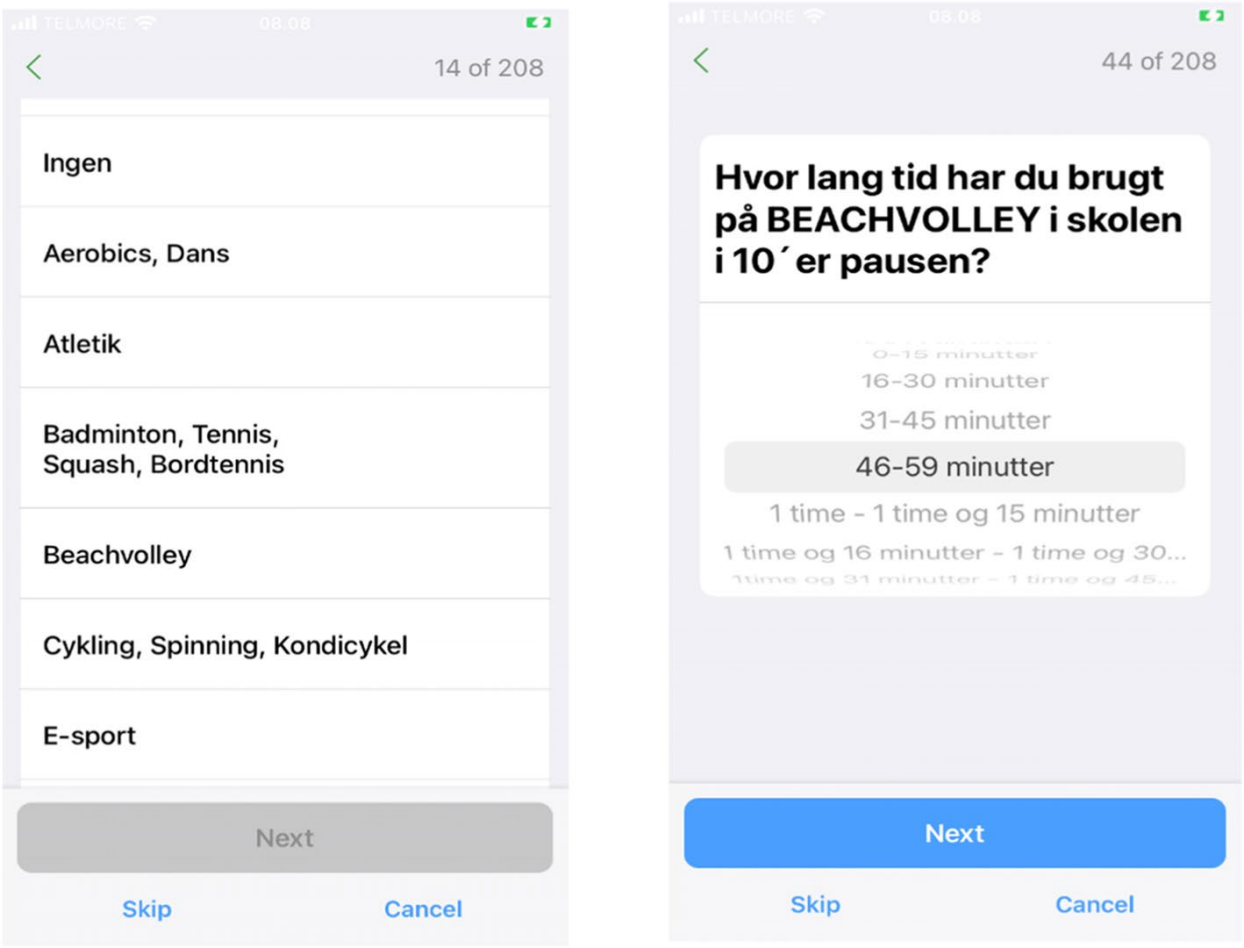
Pilot testing the Danish online version of OPAQ

Based on the preceding interviews, we made a few design changes in the online version. The week timetable from the original paper version was replaced by simple questions for each time block in the original timetable (e.g. before school, recess, before lunch, lunch, after school, evening). For example: “Which physical activities have you done during school before morning tea?” (Tick which), 33 options of activities pop up including “other” (Fig. 2). There is no limit on how many activities participants could choose. Branching logic was implemented, so that follow-up questions would appear for each activity that was selected: for example: “How much time did you spend on doing basketball” (tick: 0–15 min, 16–30 min, 31–45 min, 46–60 min, 1–1 h and 15 min, etc.). Time intervals were used to categorise the times to facilitate data collection (please refer to Fig. 2 and Additional file 3 for a visual example).

Our success criteria were that the answer response should be at least 80%, the students should wear the accelerometer at least 12 h per day in 4 out of 5 days, and 80% of the participants should fulfil these two criteria. In addition, after the data capturing period ended, the semi-structured interview was repeated to study the participants’ experience on time spent, ease of use, and success of recalling activities*.*

This online version was piloted with seven school students aged 10–13 from one Danish public school in the Region of Southern of Denmark. All students were asked to complete the Danish version of OPAQ online for five executive days while simultaneously wearing an Axivity AX3 accelerometer on their thigh for five executive days and nights [21].

The AX3 accelerometer is a small lightweight triaxial accelerometer, weighing 16 g. The accelerometer was placed on the thigh by the researcher and was mounted with plaster. It was meant to be worn 24 h per day. If the students for any reason needed to remove the accelerometer from their thigh, their parents were instructed on how to remount the accelerometer, and they received an extra set of plaster.

### Data management and analyses

#### Interviews

Data from the interviews of the students who tested the paper version of OPAQ were categorised and used to improve the online version.

#### Physical activity data

Physical activity data from the OPAQ were exported from the REDCap database into Stata 16.0 (StataCorp, TX, USA) for analysis. To quantify moderate to vigorous physical activities, any logged activities are categorised as sedentary, and light was excluded. This was done using the “Youth Compendium of Physical Activities: Activity Codes and Metabolic Intensities” [15] to categorise activities.

The accelerometer raw data were first downloaded by the OMGUI Configuration and Analysis Tool available with the Axivity AX3 into actigraph counts metric [22]. The processing of the raw data was undertaken using the software Propero Actigraph Data Analyzer V.1.1.2 (RICH, University of Southern Denmark, Denmark). Valid wear periods were identified by evaluating acceleration and temperature [23]. Time spent in moderate to vigorous intensity of physical activity was calculated using accelerometer wear time as denominator and expressed in minutes. Bland-Altman plots were conducted for describing the agreement between objective actigraph data and self-reported data (OPAQ) [24] using Stata 17.0. Due to the small sample, for the Bland-Altman LOA (limits of agreement), all observations were regarded as independent even though they come from the same student.

## Results

### Linguistic translation and cross-cultural adaptation

No major challenges were identified when translating OPAQ. Based on cultural adaptations, some of the suggested activities were added and removed in the scheme so that the list of activities was comprehensible and suitable for use in Denmark.

The translated Danish version of OPAQ was easy to understand and use, and the questions were deemed relevant. The interviews revealed a few difficulties concerning recalling activities. Furthermore, the introductory question “what is your cultural background” was found difficult by five of the students. This was replaced with two questions: “Country of birth?” and “Which languages do you primarily speak at home”. The interviews also revealed that the students forgot to note some of the PA they had done during the week. The students who received the questionnaire at the end of the week found it difficult to recall 7 days accurately, while the students who received the questionnaire in the beginning of the week and replied every evening had no difficulties.

The students asked for more space in the timetable and hints to recall activities. They found it difficult to report more than an hour when estimating the number of minutes they spent on each activity, and many did it in different ways. For all details concerning the linguistic translation and cross-cultural adaption, please refer to the supplemental (Additional file 4).

### Evaluation of the online version

The compliance was 85.7%. Seven participants completed the OPAQ for 4 out of 5 days, while two completed all 5 days. In the MyCap application, a technical error occurred 1 day and obstructed the students from completing their report on that day. Students wore the accelerometer on average 23 h and 55 min per day (Table 1).

**Table 1:** Results of self-reported physical activity (OPAQ) and objective actigraph data

OPAQ			Actigraph	
	Total minutes MVPA^a^ (min/day)^b^	Days completed	Total minutes MVPA^a^ (min/day)^b^	Wear time Days/hours
Girl 1	90 (22.25)	4	175 (35)	5/24
Girl 2	195 (39.00)	5	320 (64)	5/23.77
Girl 3	285 (71.25)	4	505 (101)	5/24
Boy 1	255 (63.75)	4	585 (117)	5/24
Boy 2	225 (56.25)	4	435 (87)	5/24
Boy 3	285 (56.25)	4	265 (53)	5/24
Boy 4	131 (26.25)	5	415 (83)	5/23.78

^a^ Moderate to vigorous physical activity. ^b^ Average minutes per day

**Fig. 3:**
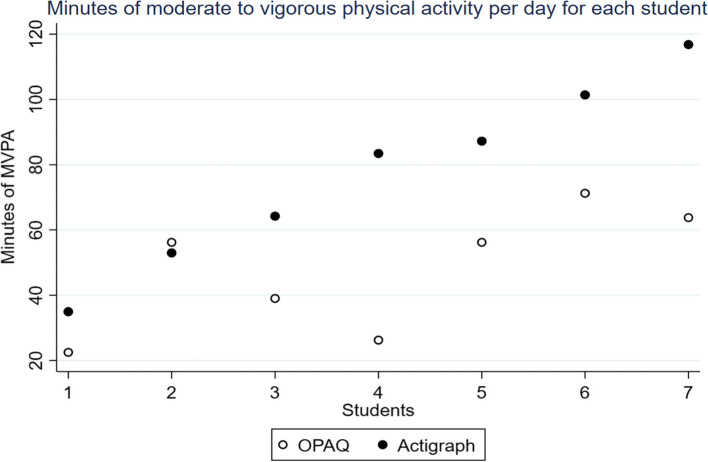
Scatterplot of self-reported physical activity and objective actigraph captured physical activity in average per day

**Fig. 4:**
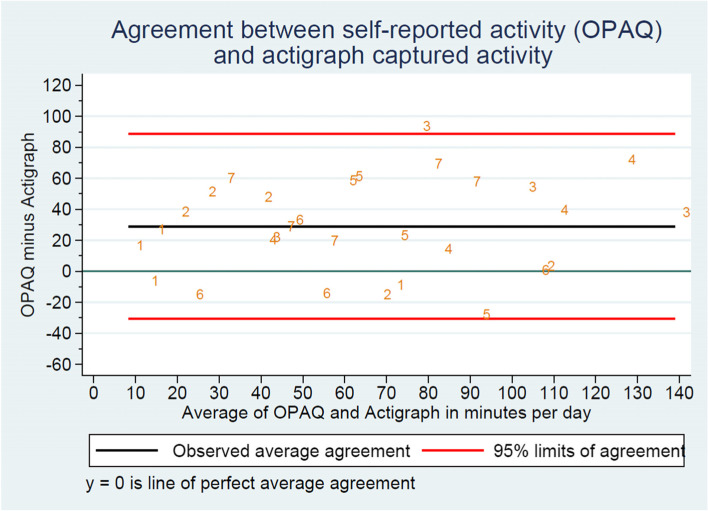
Bland-Altman plot illustrating the agreement between self-reported daily physical activity using the Oxford Physical Activity Questionnaire and objective captured actigraph physical activity

Regarding time spent in moderate to vigorous physical activity as measured by the accelerometer versus the self-report, there was good agreement measured as difference in minutes per day. Six of the seven students reported slightly less activity (range 10–60 min) compared to the actigraph measures. One student reported 3 min more (Table 1, Figs. 3 and 4). A single observation lies outside the 95% limits of agreement (Fig. 4). The primary activities the students reported were trampoline, tumbling gymnastics, skating, soccer, walking, and TikTok dancing.

The interviews revealed that the app was easy to use and the questionnaire was easy to understand for the respondents and quick to fill in. Due to the specific questions and the daily design, the participants experienced it easy to recall their activities.

## Discussion

The online version of the OPAQ was feasible for secondary school students to complete, and the MyCap app was user-friendly for data capturing. There was good agreement between the objective actigraph data (golden standard) and the OPAQ. As would be expected, there was a slight tendency for lower moderate to vigorous physical activity recorded by the OPAQ compared to the accelerometer.

The reason for this is likely due to the accelerometer capturing spontaneous movement which the school students would not categorise as sport of physical activity. For example, minor activities such as running up the stairs or in the playground and jumping are not likely logged by students but will be recorded by the accelerometers. This is because the thresholds for moderate to vigorous physical activity were based on counts per minute only, and not bouts of any minimum duration. Previous studies found that participants (youth and adults) tend to overreport moderate to vigorous physical activity compared to the objective measured activity [25–27].

To our knowledge, no previous studies have compared paper-based questionnaires data and smartphone-based questionnaires data in secondary school students. One study among adults compared paper-based data collection with an electronic data collection system [28]. It was reported that the electronic version produced fewer missing data, fewer errors and inconsistent responses, and delivered data faster. Using smartphones therefore has potential to improve timeliness and data integrity and reduce costs [28]. However, it requires that mobile phones are integrated in the school students’ everyday life, and that most school students have a smartphone. Using smartphones makes it possible and easy to capture data every day which limits the amount of memory decay that can take place over time. Previous studies have reported high compliance when using mobile phones for data collection in youth and adults [29, 30].

In our study, we observed similar findings. In the paper version, the school students needed to write and spell every single answer which for some were difficult. It also gave some challenges in managing, cleaning, and processing the data. It could be hard to read and understand, and it was time demanding to recode and analyse the collected data. The online data capture reduces the potential for errors and is a secure way to manage data. During the data collecting period, it is possible to monitor if respondents record their activity and provide reminders if they do not. In the paper version, the students must estimate the number of minutes they spent on each activity, but they found it difficult to report more than an hour, and many did it in different ways. Therefore, we included standardised time intervals in the online version. This could be potentially changed by replacing the time intervals with drop-down boxes including numbers of hours and numbers of minutes.

To minimise recall bias, we chose to ask the students to report every day. Therefore, we included the demographic questions in our baseline questionnaire the students completed when volunteering for the study. Compared to both the paper version and objective measures this online version makes, it very easy to repeat capturing physical activity data during a study period. The questionnaire will pop up automatically on the students’ devices.

This study has some limitations. The primary aim to convert the original paper version to an online Danish version was to develop an already validated physical activity questionnaire to a more user-friendly. There were problems with recruiting a larger sample as intended due to COVID-19 lockdown, which impeded a full validation.

Overall, the students responded that the OPAQ was easy and quick to complete but with the first day being the most time demanding. We did not investigate if the paper version and the electronic version may potentially yield different outcomes in the same group of students. However, the interviews revealed the students were much happier and more compliant with the online version versus the paper version.

Due to the few students in this study, there may be a risk of selection bias, if students who have an interest in physical activity were more likely to participate. This could influence the compliance of the daily completion of OPAQ. However, all students were included from school’s population, and it is not clear if any selection bias occurred.

In conclusion, the online version of the Oxford Physical Activity Questionnaire was perceived as quick and easy for the students to complete during a 1-week period. The daily design was found helpful for remembering physical activity compared to the original 1-week recall. Using MyCap app on the students’ smartphones as data capturing tool was a success. In previous studies, normal procedure has been to let the parents report their children’s activity level, but in this study, the students’ age 10–13 showed that they are able to do so by themselves when smartphones are used as data capturing tool. We find the Danish online version of OPAQ useful in cohort and survey studies, keeping in mind that the OPAQ tend to underreport the students’ daily moderate to vigorous physical activity level. As the design of the online version was changed compared to the original paper version, examination of the convergent validity and test-retest reliability of this version are needed.

## Supplementary Information

**Additional file 1.** The Oxford Physical Activity Questionnaire (OPAQ).

**Additional file 2.** Baseline questionnaire.

**Additional file 3.** The online version of OPAQ.

**Additional file 4.** Linguistic translation and cross-cultural adaptation.

## Data Availability

The datasets generated and analysed during the current study are available from the corresponding author on reasonable request.
