# Supplementary material for: Adaptation of a Danish online version of the Oxford Physical Activity Questionnaire (OPAQ) for secondary school students—a pilot study
Source: Pilot Feasibility Stud. 2022 Jul 25;8:153. doi: 10.1186/s40814-022-01108-x (PMC9309605; doi:10.1186/s40814-022-01108-x)
Supplement: Supplementary file 1 — Additional file 1. The Oxford Physical Activity Questionnaire (OPAQ). [file 40814_2022_1108_MOESM1_ESM.pdf]

Appendix 1: The Oxford Physical Activity Questionnaire (OPAQ)

Oxford Physical Activity Questionnaire (OPAQ)

Student Name: ______________________________

School: ____________________________________

To protect your privacy this cover sheet will be removed and destroyed once You've been allocated a study number.

Oxford Physical Activity Questionnaire (OPAQ)

1) **Age:** _______

2) **Gender:** *please tick* (✓) ◻ Male ◻ Female

3) **Height:** ______cm **Weight:** ______kg

4) **In what country were you born?** *Please tick* (✓)
◻ Australia ◻ Another country (Please specify): ____________________

5) **What is your cultural background?**
◻ Australian ◻ Aboriginal ◻ Torres Strait Islander ◻ Asian ◻ European
◻ Middle Eastern ◻ African ◻ Other: (please specify) ____________

6) **What language do you speak most at home?** *Please tick* (✓)
◻ **English** ◻ **Another language - (please specify):** ________________________

7) **What is the postcode of the suburb you live in?** __ __ __ __

The purpose of this questionnaire is to estimate the amount of time you spent participating in physical activity over the past 7 days. This includes physical education, school sport and other moderate to vigorous physical activity you completed during the week and on the weekend.

Moderate to vigorous physical activity makes you breathe heavily and increases your heart rate. It includes all sports, exercise activities, games, swimming, running, skateboarding etc.

Please indicate the **ACTIVITY** and the **TIME** you spent doing the activity. There are a number of questions that will help you to remember, along with a list of common activities at the top of the next page. If you did not do any physical activity, please leave the table blank.

1) **How do you usually travel to school?** (*Please circle)*

Walk *Cycle* *Bus* *Car* *Other*

2) How long does each journey take? *(Write)* _______________ mins.

3) Over the past 7 days, on how many days did you walk or ride, scoot or skate to school? *(Please circle)*.

0 1 2 3 4 5 6

<u>Now, please record the following information on the timetable on the NEXT PAGE:</u>

4) List all the practical activities that you did in PE lessons over the past 7 days (e.g. football, hockey, dance, and gymnastics).

5) List all the activities that you did in school sport over the past 7 days (e.g. basketball, weight training, and golf).

6) List any sports or activities that you did before/after school or on weekends over the past 7 days (e.g. surfing, cricket and dance).

7) List any other physical activities that you did over the past 7 days that lasted 20 minutes or longer (e.g. you might have played soccer at recess or lunchtime).

Oxford Physical Activity Questionnaire

Aerobics	Cycling	Hockey	Netball	Soccer
Athletics	Dance (ballet)	Indoor soccer	Rowing	Surfing
Austag	Dance (ballroom)	Inline hockey	Running (jogging)	Swimming
Baseball	Dance (jazz)	Inline skating	Rugby league	Tennis
Basketball	Golf	Lifesaving	Rugby union	Touch football
Cricket	Gymnastics	Martial arts	Skateboarding	Volleyball

<table>
<tr><th colspan="15">Participation in physical activity during the last 7 days</th></tr>
<tr><th rowspan="2">Component</th><th colspan="2">Monday</th><th colspan="2">Tuesday</th><th colspan="2">Wednesday</th><th colspan="2">Thursday</th><th colspan="2">Friday</th><th colspan="2">Saturday</th><th colspan="2">Sunday</th></tr>
<tr><th>Activity</th><th>Mins</th><th>Activity</th><th>Mins</th><th>Activity</th><th>Mins</th><th>Activity</th><th>Mins</th><th>Activity</th><th>Mins</th><th>Activity</th><th>Mins</th><th>Activity</th><th>Mins</th></tr>
<tr><td>Before school</td><td></td><td></td><td></td><td></td><td></td><td></td><td></td><td></td><td></td><td></td><td rowspan="6">Surfing</td><td rowspan="6">120</td><td rowspan="6">Touch football</td><td rowspan="6">60</td></tr>
<tr><td>Morning
(including breaks)</td><td></td><td></td><td></td><td></td><td></td><td></td><td>Dance(PE)</td><td>40</td><td></td><td></td></tr>
<tr><td>Lunch time</td><td></td><td></td><td>Basketball</td><td>45</td><td></td><td></td><td></td><td></td><td></td><td></td></tr>
<tr><td>Afternoon
(including breaks)</td><td></td><td></td><td></td><td></td><td></td><td></td><td></td><td></td><td></td><td></td></tr>
<tr><td>After school</td><td></td><td></td><td></td><td></td><td></td><td></td><td></td><td></td><td>Soccer</td><td>90</td></tr>
<tr><td>Evening</td><td></td><td></td><td></td><td></td><td></td><td></td><td></td><td></td><td></td><td></td></tr>
<tr><td colspan="15">Note: Remember to include the number of minutes, examples have been have been provided for you.</td></tr>
</table>
