# Supplementary material for: Adaptation of a Danish online version of the Oxford Physical Activity Questionnaire (OPAQ) for secondary school students—a pilot study
Source: Pilot Feasibility Stud. 2022 Jul 25;8:153. doi: 10.1186/s40814-022-01108-x (PMC9309605; doi:10.1186/s40814-022-01108-x)
Supplement: Supplementary file 2 — Additional file 2. Baseline questionnaire. [file 40814_2022_1108_MOESM2_ESM.pdf]

Tilmelding

Venligst udfyld nedenstående spørgeskema

Tak!

Inden du kan deltage i denne undersøgelse, skal du og dine forældre bekræfte, at I har læst den tilsendte deltagerinformation grundigt igennem sammen og at du samtykker til deltagelse.

Afkryds venligst følgende:

Jeg forstår, at deltagelse i projektet er frivilligt og at jeg til en hver tid kan stoppe med at deltage i undersøgelsen	◯ Ja
Jeg forstår, at oplysninger indsamlet gennem denne undersøgelse vil blive behandlet fortroligt, at oplysningerne kun vil blive brugt til forskning og at formidling af forskningsresultaterne vil ske i anonymiseret form	◯ Ja
Jeg giver tilladelse til, at de data, der indsamles fra denne undersøgelse må bruges til forskning og at disse data vil blive delt med internationale forskningspartnere, der er involveret i forskningsprojektet	◯ Ja

VELKOMMEN

I de følgende spørgsmål vil der være spørgsmål både du og dine forældre skal svare på.

Det tager højest 5 minutter at besvare.

Fornavn Mellemnavn(e)	________________
Efternavn	________________
E-mail adresse?	________________
CPR nummer	________________<br>(Cpr nummer med bindestreg)
Køn	◯ Dreng<br>◯ Pige
Vægt i kg	________________<br>(Skriv kun hele tal - f.eks. 28 eller 65)

Højde i cm	____________________<br>(Skriv kun hele tal - f.eks. 154)
BMI	____________________
I hvilket land er du født?	◯ Danmark<br>◯ Andet
Hvilket?	____________________
Hvilket sprog taler du mest hjemme?	◯ Dansk<br>◯ Andet
Hvilket?	____________________
Hvilket postnummer bor du i?	____________________
Hvilken skole går du på?	◯ Fælleshåbsskolen, fælleshåbsvej<br>◯ Englystskolen
Hvilken klasse går du i?	◯ 4.a<br>◯ 4.b<br>◯ 4.c<br>◯ 4.d<br>◯ 5.a<br>◯ 5.b<br>◯ 5.c<br>◯ 5.d<br>◯ 6.a<br>◯ 6.b<br>◯ 6.c<br>◯ 6.d<br>◯ 7.a<br>◯ 7.b<br>◯ 7.c<br>◯ 7.d<br>◯ 8.a<br>◯ 8.b<br>◯ 8.c<br>◯ 8.d<br>◯ 9.a<br>◯ 9.b<br>◯ 9.c<br>◯ 9.d
Hvordan kommer du oftest til og fra skole?	◯ Går<br>◯ Cykler<br>◯ Køre i bil<br>◯ Køre i bus<br>◯ Køre i tog<br>◯ Andet
Hvordan?	____________________

Går du til sport i din fritid?	◯ Ja<br>◯ Nej
Hvilken/hvilke?	☐ Aerobics, Dans<br>☐ Atletik<br>☐ Badminton, Tennis, Squash, Bordtennis<br>☐ Ballet<br>☐ Beachvolley<br>☐ Cykling, Spinning, Kondicykel<br>☐ E-sport, computerspil<br>☐ Floorball<br>☐ Fodbold<br>☐ Golf<br>☐ Gymnastik, Rytme, Spring, Tumbling<br>☐ Håndbold, Basketball, Hockey<br>☐ Ishockey, Is-skøjteløb<br>☐ Kano, Kajak, Roning<br>☐ Kampsport (f.eks. karate, boksning, taekwondo<br>☐ Løb<br>☐ Parkour<br>☐ Ridning<br>☐ Rugby, Amerikansk fodbold<br>☐ Sejlsport<br>☐ Skateboard, Løbehjul, Rulleskøjter<br>☐ Styrketræning<br>☐ Svømning<br>☐ Trampolin<br>☐ Volleyball<br>☐ Andet
Hvilken?	__________________________________
Hvor mange gange om ugen går du til sport?	◯ 0<br>◯ 1-2<br>◯ 3-4<br>◯ 5 eller flere
Har du plaster allergi?	◯ Ja<br>◯ Nej
Bor dine forældre sammen?	◯ Ja<br>◯ Nej
Bor du sammen med dine forældre?	◯ Ja<br>◯ Nej
Hvem bor du hos?	◯ Mor<br>◯ Far<br>◯ Lige mange dage hos mor/far<br>◯ Andet
Hvor eller hvem bor du hos?	__________________________________<br>(F.eks. bedsteforældre, plejeforældre, børnehjem)

De følgende spørgsmål er til din/dine forældre

Far

Højeste uddannelseniveau

- ◯ Folkeskolen
- ◯ Erhvervsfaglig uddannelse
- ◯ Gymnasial uddannelse (almen, teknisk, handel, erhverv)
- ◯ Kort videregående uddannelse (under 3 år)
- ◯ Mellemlang videregående uddannelse (3-4 år)
- ◯ Lang videregående uddannelse (5- år)

Mor

Højeste uddannelsesniveau

- ◯ Folkeskolen
- ◯ Erhvervsfaglig uddannelse
- ◯ Gymnasial uddannelse (almen, teknisk, handel, erhverv)
- ◯ Kort videregående uddannelse (under 3 år)
- ◯ Mellemlang videregående uddannelse (3-4 år)
- ◯ Lang videregående uddannelse (5- år)

Far

- ◯ I arbejde
- ◯ Ikke i arbejde

Sygemeldt?

- ◯ Ja
- ◯ Nej
- ◯ Ønsker ikke at svare

Mor

- ◯ I arbejde
- ◯ Ikke i arbejde

Sygemeldt?

- ◯ Ja
- ◯ Nej
- ◯ Ønsker ikke at svare

På barsel?

- ◯ Ja
- ◯ Nej

Far

- ◯ Dyrker sport
- ◯ Dyrker ikke sport

Hvor mange gange om ugen dyrker du sport?

- ◯ 0
- ◯ 1-2
- ◯ 3-4
- ◯ 5-

Mor

- ◯ Dyrker sport
- ◯ Dyrker ikke sport

Hvor mange gange om ugen dyrker du sport?

- ◯ 0
- ◯ 1-2
- ◯ 3-4
- ◯ 5-

Felter til styring af Baseline dato og MyCap generelt (vises kun i designeren)

Gruppe 1 dato ______________________________

Gruppe 2 dato ______________________________

Gruppe 3 dato ______________________________

Gruppeberegner ______________________________

Baseline Date ______________________________

Code ______________________________

Install Date ______________________________

Push Notification IDs ______________________________
