# Supplementary material for: Adaptation of a Danish online version of the Oxford Physical Activity Questionnaire (OPAQ) for secondary school students—a pilot study
Source: Pilot Feasibility Stud. 2022 Jul 25;8:153. doi: 10.1186/s40814-022-01108-x (PMC9309605; doi:10.1186/s40814-022-01108-x)
Supplement: Supplementary file 3 — Additional file 3. The online version of OPAQ. [file 40814_2022_1108_MOESM3_ESM.pdf]

Opaq

Record ID ______________________________

I de næste spørgsmål skal du beskrive hvilke fysiske aktiviteter, du har lavet før, efter og i skolen i dag.

Husk at notere alle de AKTIVITETER du har lavet og i hvor lang TID du har lavet dem.

Hvilke fysiske aktiviteter har du lavet inden du mødte i skole i dag?

- ☐ Ingen
- ☐ Cyklet til skole
- ☐ Gået til skole
- ☐ Løbehjul, skateboard, rulleskøjter til skole
- ☐ Atletik
- ☐ Cykling, Spinning, Kondicykel (som træning ikke transport til skole)
- ☐ Styrketræning
- ☐ Svømning
- ☐ Løb
- ☐ Trampolin
- ☐ Gå tur (ikke som transport til skole)
- ☐ Andet

Hvor lang tid har du brugt på at CYKLE til skole?

- ◯ 0-15 minutter
- ◯ 16-30 minutter
- ◯ 31-45 minutter
- ◯ 46-59 minutter
- ◯ 1 time - 1 time og 15 minutter
- ◯ 1 time og 16 minutter - 1 time og 30 minutter
- ◯ 1time og 31 minutter - 1 time og 45 minutter
- ◯ 1time og 46 minutter - 2 timer
- ◯ Mere end 2 timer

Hvor lang tid har du brugt på at GÅ til skole?

- ◯ 0-15 minutter
- ◯ 16-30 minutter
- ◯ 31-45 minutter
- ◯ 46-59 minutter
- ◯ 1 time - 1 time og 15 minutter
- ◯ 1 time og 16 minutter - 1 time og 30 minutter
- ◯ 1time og 31 minutter - 1 time og 45 minutter
- ◯ 1time og 46 minutter - 2 timer
- ◯ Mere end 2 timer

Hvor lang tid har du brugt på at SKATE, køre på RULLESKØJTER eller LØBEHJUL til skole?

- ◯ 0-15 minutter
- ◯ 16-30 minutter
- ◯ 31-45 minutter
- ◯ 46-59 minutter
- ◯ 1 time - 1 time og 15 minutter
- ◯ 1 time og 16 minutter - 1 time og 30 minutter
- ◯ 1time og 31 minutter - 1 time og 45 minutter
- ◯ 1time og 46 minutter - 2 timer
- ◯ Mere end 2 timer

Hvor lang tid har du brugt på ATLETIK, inden du mødte i skole?	◯ 0-15 minutter<br>◯ 16-30 minutter<br>◯ 31-45 minutter<br>◯ 46-59 minutter<br>◯ 1 time - 1 time og 15 minutter<br>◯ 1 time og 16 minutter - 1 time og 30 minutter<br>◯ 1time og 31 minutter - 1 time og 45 minutter<br>◯ 1time og 46 minutter - 2 timer<br>◯ Mere end 2 timer
Hvor lang tid har du brugt på STYRKETRÆNING, inden du mødte i skole?	◯ 0-15 minutter<br>◯ 16-30 minutter<br>◯ 31-45 minutter<br>◯ 46-59 minutter<br>◯ 1 time - 1 time og 15 minutter<br>◯ 1 time og 16 minutter - 1 time og 30 minutter<br>◯ 1time og 31 minutter - 1 time og 45 minutter<br>◯ 1time og 46 minutter - 2 timer<br>◯ Mere end 2 timer
Hvor lang tid har du brugt på SVØMNING, inden du mødte i skole?	◯ 0-15 minutter<br>◯ 16-30 minutter<br>◯ 31-45 minutter<br>◯ 46-59 minutter<br>◯ 1 time - 1 time og 15 minutter<br>◯ 1 time og 16 minutter - 1 time og 30 minutter<br>◯ 1time og 31 minutter - 1 time og 45 minutter<br>◯ 1time og 46 minutter - 2 timer<br>◯ Mere end 2 timer
Hvor lang tid har du brugt på CYKLING, SPINNING, KONDICYKEL, inden du mødte i skole?	◯ 0-15 minutter<br>◯ 16-30 minutter<br>◯ 31-45 minutter<br>◯ 46-59 minutter<br>◯ 1 time - 1 time og 15 minutter<br>◯ 1 time og 16 minutter - 1 time og 30 minutter<br>◯ 1time og 31 minutter - 1 time og 45 minutter<br>◯ 1time og 46 minutter - 2 timer<br>◯ Mere end 2 timer
Hvor lang tid har du brugt på at løbe, inden du mødte i skole?	◯ 0-15 minutter<br>◯ 16-30 minutter<br>◯ 31-45 minutter<br>◯ 46-59 minutter<br>◯ 1 time - 1 time og 15 minutter<br>◯ 1 time og 16 minutter - 1 time og 30 minutter<br>◯ 1time og 31 minutter - 1 time og 45 minutter<br>◯ 1time og 46 minutter - 2 timer<br>◯ Mere end 2 timer
Hvor lang tid har du brugt på at HOPPE I TRAMPOLIN, inden du mødte i skole?	◯ 0-15 minutter<br>◯ 16-30 minutter<br>◯ 31-45 minutter<br>◯ 46-59 minutter<br>◯ 1 time - 1 time og 15 minutter<br>◯ 1 time og 16 minutter - 1 time og 30 minutter<br>◯ 1time og 31 minutter - 1 time og 45 minutter<br>◯ 1time og 46 minutter - 2 timer<br>◯ Mere end 2 timer

Hvor lang tid har du brugt på at GÅ EN TUR, inden du mødte i skole?	◯ 0-15 minutter<br>◯ 16-30 minutter<br>◯ 31-45 minutter<br>◯ 46-59 minutter<br>◯ 1 time - 1 time og 15 minutter<br>◯ 1 time og 16 minutter - 1 time og 30 minutter<br>◯ 1time og 31 minutter - 1 time og 45 minutter<br>◯ 1time og 46 minutter - 2 timer<br>◯ Mere end 2 timer
Hvilken anden fysisk aktivitet har du lavet?	______________________________
Hvor lang tid har du brugt på denne aktivitet?	◯ 0-15 minutter<br>◯ 16-30 minutter<br>◯ 31-45 minutter<br>◯ 46-59 minutter<br>◯ 1 time - 1 time og 15 minutter<br>◯ 1 time og 16 minutter - 1 time og 30 minutter<br>◯ 1time og 31 minutter - 1 time og 45 minutter<br>◯ 1time og 46 minutter - 2 timer<br>◯ Mere end 2 timer
Hvilke fysiske aktivieter har du lavet i timerne før din 10´er pause i dag?	☐ Ingen<br>☐ Aerobics, Dans<br>☐ Atletik<br>☐ Badminton, Tennis, Squash, Bordtennis<br>☐ Beachvolley<br>☐ Cykling, Spinning, Kondicykel<br>☐ E-sport<br>☐ Floorball<br>☐ Fodbold<br>☐ Gemmeleg, Firkant, Spark til dåsen, Fangeleg/Tik<br>☐ Golf<br>☐ Gymnastik, Rytme, Spring, Tumbling<br>☐ Gåtur<br>☐ Håndbold, Basketball, Hockey<br>☐ Høvdingebold, Rundbold<br>☐ Is-skøjteløb, Ishockey<br>☐ Kampsport (f.eks. karate, boksning, taekwondo)<br>☐ Løb<br>☐ Parkour<br>☐ Rugby, Amerikansk fodbold<br>☐ Skateboard, Løbehjul, Rulleskøjter<br>☐ Styrketræning<br>☐ Svømning<br>☐ Volleyball<br>☐ Andet
Hvor lang tid har du brugt på AEROBICS, DANS i skolen før 10´er pausen?	◯ 0-15 minutter<br>◯ 16-30 minutter<br>◯ 31-45 minutter<br>◯ 46-59 minutter<br>◯ 1 time - 1 time og 15 minutter<br>◯ 1 time og 16 minutter - 1 time og 30 minutter<br>◯ 1time og 31 minutter - 1 time og 45 minutter<br>◯ 1time og 46 minutter - 2 timer<br>◯ Mere end 2 timer

Hvor lang tid har du brugt på ATLETIK i skolen før 10´er pausen?	◯ 0-15 minutter<br>◯ 16-30 minutter<br>◯ 31-45 minutter<br>◯ 46-59 minutter<br>◯ 1 time - 1 time og 15 minutter<br>◯ 1 time og 16 minutter - 1 time og 30 minutter<br>◯ 1time og 31 minutter - 1 time og 45 minutter<br>◯ 1time og 46 minutter - 2 timer<br>◯ Mere end 2 timer
Hvor lang tid har du brugt på BADMINTON, TENNIS, SQUASH, BORDTENNIS i skolen før 10´er pausen?	◯ 0-15 minutter<br>◯ 16-30 minutter<br>◯ 31-45 minutter<br>◯ 46-59 minutter<br>◯ 1 time - 1 time og 15 minutter<br>◯ 1 time og 16 minutter - 1 time og 30 minutter<br>◯ 1time og 31 minutter - 1 time og 45 minutter<br>◯ 1time og 46 minutter - 2 timer<br>◯ Mere end 2 timer
Hvor lang tid har du brugt på BEACHVOLLEY i skolen før 10´er pausen?	◯ 0-15 minutter<br>◯ 16-30 minutter<br>◯ 31-45 minutter<br>◯ 46-59 minutter<br>◯ 1 time - 1 time og 15 minutter<br>◯ 1 time og 16 minutter - 1 time og 30 minutter<br>◯ 1time og 31 minutter - 1 time og 45 minutter<br>◯ 1time og 46 minutter - 2 timer<br>◯ Mere end 2 timer
Hvor lang tid har du brugt på CYKLING, SPINNING, KONDICYKEL i skolen før 10´er pausen?	◯ 0-15 minutter<br>◯ 16-30 minutter<br>◯ 31-45 minutter<br>◯ 46-59 minutter<br>◯ 1 time - 1 time og 15 minutter<br>◯ 1 time og 16 minutter - 1 time og 30 minutter<br>◯ 1time og 31 minutter - 1 time og 45 minutter<br>◯ 1time og 46 minutter - 2 timer<br>◯ Mere end 2 timer
Hvor lang tid har du brugt på E-SPORT i skolen før 10´er pausen?	◯ 0-15 minutter<br>◯ 16-30 minutter<br>◯ 31-45 minutter<br>◯ 46-59 minutter<br>◯ 1 time - 1 time og 15 minutter<br>◯ 1 time og 16 minutter - 1 time og 30 minutter<br>◯ 1time og 31 minutter - 1 time og 45 minutter<br>◯ 1time og 46 minutter - 2 timer<br>◯ Mere end 2 timer
Hvor lang tid har du brugt på FLOORBALL i skolen før 10´er pausen?	◯ 0-15 minutter<br>◯ 16-30 minutter<br>◯ 31-45 minutter<br>◯ 46-59 minutter<br>◯ 1 time - 1 time og 15 minutter<br>◯ 1 time og 16 minutter - 1 time og 30 minutter<br>◯ 1time og 31 minutter - 1 time og 45 minutter<br>◯ 1time og 46 minutter - 2 timer<br>◯ Mere end 2 timer

Hvor lang tid har du brugt på FODBOLD i skolen før 10´er pausen?	◯ 0-15 minutter<br>◯ 16-30 minutter<br>◯ 31-45 minutter<br>◯ 46-59 minutter<br>◯ 1 time - 1 time og 15 minutter<br>◯ 1 time og 16 minutter - 1 time og 30 minutter<br>◯ 1time og 31 minutter - 1 time og 45 minutter<br>◯ 1time og 46 minutter - 2 timer<br>◯ Mere end 2 timer
Hvor lang tid har du brugt på GEMMELEG, FIRKANT, SPARK TIL DÅSEN, FANGELEG/TIK i skolen før 10´er pausen?	◯ 0-15 minutter<br>◯ 16-30 minutter<br>◯ 31-45 minutter<br>◯ 46-59 minutter<br>◯ 1 time - 1 time og 15 minutter<br>◯ 1 time og 16 minutter - 1 time og 30 minutter<br>◯ 1time og 31 minutter - 1 time og 45 minutter<br>◯ 1time og 46 minutter - 2 timer<br>◯ Mere end 2 timer
Hvor lang tid har du brugt på GOLF i skolen før 10´er pausen?	◯ 0-15 minutter<br>◯ 16-30 minutter<br>◯ 31-45 minutter<br>◯ 46-59 minutter<br>◯ 1 time - 1 time og 15 minutter<br>◯ 1 time og 16 minutter - 1 time og 30 minutter<br>◯ 1time og 31 minutter - 1 time og 45 minutter<br>◯ 1time og 46 minutter - 2 timer<br>◯ Mere end 2 timer
Hvor lang tid har du brugt på GYMNASTIK i skolen før 10´er pausen?	◯ 0-15 minutter<br>◯ 16-30 minutter<br>◯ 31-45 minutter<br>◯ 46-59 minutter<br>◯ 1 time - 1 time og 15 minutter<br>◯ 1 time og 16 minutter - 1 time og 30 minutter<br>◯ 1time og 31 minutter - 1 time og 45 minutter<br>◯ 1time og 46 minutter - 2 timer<br>◯ Mere end 2 timer
Hvor lang tid har du brugt på GÅTUR i skolen før 10´er pausen?	◯ 0-15 minutter<br>◯ 16-30 minutter<br>◯ 31-45 minutter<br>◯ 46-59 minutter<br>◯ 1 time - 1 time og 15 minutter<br>◯ 1 time og 16 minutter - 1 time og 30 minutter<br>◯ 1time og 31 minutter - 1 time og 45 minutter<br>◯ 1time og 46 minutter - 2 timer<br>◯ Mere end 2 timer
Hvor lang tid har du brugt på HÅNDBOLD, BASKETBALL, HOCKEY i skolen før 10´er pausen?	◯ 0-15 minutter<br>◯ 16-30 minutter<br>◯ 31-45 minutter<br>◯ 46-59 minutter<br>◯ 1 time - 1 time og 15 minutter<br>◯ 1 time og 16 minutter - 1 time og 30 minutter<br>◯ 1time og 31 minutter - 1 time og 45 minutter<br>◯ 1time og 46 minutter - 2 timer<br>◯ Mere end 2 timer

Hvor lang tid har du brugt på HØVDINGEBOLD, RUNDBOLD i skolen før 10´er pausen?	◯ 0-15 minutter<br>◯ 16-30 minutter<br>◯ 31-45 minutter<br>◯ 46-59 minutter<br>◯ 1 time - 1 time og 15 minutter<br>◯ 1 time og 16 minutter - 1 time og 30 minutter<br>◯ 1time og 31 minutter - 1 time og 45 minutter<br>◯ 1time og 46 minutter - 2 timer<br>◯ Mere end 2 timer
Hvor lang tid har du brugt på IS-SKØJTELØB, ISHOCKEY i skolen før 10´er pausen?	◯ 0-15 minutter<br>◯ 16-30 minutter<br>◯ 31-45 minutter<br>◯ 46-59 minutter<br>◯ 1 time - 1 time og 15 minutter<br>◯ 1 time og 16 minutter - 1 time og 30 minutter<br>◯ 1time og 31 minutter - 1 time og 45 minutter<br>◯ 1time og 46 minutter - 2 timer<br>◯ Mere end 2 timer
Hvor lang tid har du brugt på KAMPSPORT (f.eks. karate, boksning, teak wondo) i skolen før 10´er pausen?	◯ 0-15 minutter<br>◯ 16-30 minutter<br>◯ 31-45 minutter<br>◯ 46-59 minutter<br>◯ 1 time - 1 time og 15 minutter<br>◯ 1 time og 16 minutter - 1 time og 30 minutter<br>◯ 1time og 31 minutter - 1 time og 45 minutter<br>◯ 1time og 46 minutter - 2 timer<br>◯ Mere end 2 timer
Hvor lang tid har du brugt på LØB i skolen før 10´er pausen?	◯ 0-15 minutter<br>◯ 16-30 minutter<br>◯ 31-45 minutter<br>◯ 46-59 minutter<br>◯ 1 time - 1 time og 15 minutter<br>◯ 1 time og 16 minutter - 1 time og 30 minutter<br>◯ 1time og 31 minutter - 1 time og 45 minutter<br>◯ 1time og 46 minutter - 2 timer<br>◯ Mere end 2 timer
Hvor lang tid har du brugt på PARKOUR i skolen før 10´er pausen?	◯ 0-15 minutter<br>◯ 16-30 minutter<br>◯ 31-45 minutter<br>◯ 46-59 minutter<br>◯ 1 time - 1 time og 15 minutter<br>◯ 1 time og 16 minutter - 1 time og 30 minutter<br>◯ 1time og 31 minutter - 1 time og 45 minutter<br>◯ 1time og 46 minutter - 2 timer<br>◯ Mere end 2 timer
Hvor lang tid har du brugt på Rugby, Amerikansk fodbold i skolen før 10´er pausen?	◯ 0-15 minutter<br>◯ 16-30 minutter<br>◯ 31-45 minutter<br>◯ 46-59 minutter<br>◯ 1 time - 1 time og 15 minutter<br>◯ 1 time og 16 minutter - 1 time og 30 minutter<br>◯ 1time og 31 minutter - 1 time og 45 minutter<br>◯ 1time og 46 minutter - 2 timer<br>◯ Mere end 2 timer

Hvor lang tid har du brugt på SKATEBOARD, LØBEHJUL, RULLESKØJTER i skolen før 10´er pausen?	◯ 0-15 minutter<br>◯ 16-30 minutter<br>◯ 31-45 minutter<br>◯ 46-59 minutter<br>◯ 1 time - 1 time og 15 minutter<br>◯ 1 time og 16 minutter - 1 time og 30 minutter<br>◯ 1time og 31 minutter - 1 time og 45 minutter<br>◯ 1time og 46 minutter - 2 timer<br>◯ Mere end 2 timer
Hvor lang tid har du brugt på STYRKETRÆNING i skolen før 10´er pausen?	◯ 0-15 minutter<br>◯ 16-30 minutter<br>◯ 31-45 minutter<br>◯ 46-59 minutter<br>◯ 1 time - 1 time og 15 minutter<br>◯ 1 time og 16 minutter - 1 time og 30 minutter<br>◯ 1time og 31 minutter - 1 time og 45 minutter<br>◯ 1time og 46 minutter - 2 timer<br>◯ Mere end 2 timer
Hvor lang tid har du brugt på SVØMNING i skolen før 10´er pausen?	◯ 0-15 minutter<br>◯ 16-30 minutter<br>◯ 31-45 minutter<br>◯ 46-59 minutter<br>◯ 1 time - 1 time og 15 minutter<br>◯ 1 time og 16 minutter - 1 time og 30 minutter<br>◯ 1time og 31 minutter - 1 time og 45 minutter<br>◯ 1time og 46 minutter - 2 timer<br>◯ Mere end 2 timer
Hvor lang tid har du brugt på VOLLEYBALL i skolen før 10´er pausen?	◯ 0-15 minutter<br>◯ 16-30 minutter<br>◯ 31-45 minutter<br>◯ 46-59 minutter<br>◯ 1 time - 1 time og 15 minutter<br>◯ 1 time og 16 minutter - 1 time og 30 minutter<br>◯ 1time og 31 minutter - 1 time og 45 minutter<br>◯ 1time og 46 minutter - 2 timer<br>◯ Mere end 2 timer
Hvilken anden fysisk aktivitet har du lavet?	__________________________________
Hvor lang tid har du brugt på denne aktivitet?	◯ 0-15 minutter<br>◯ 16-30 minutter<br>◯ 31-45 minutter<br>◯ 46-59 minutter<br>◯ 1 time - 1 time og 15 minutter<br>◯ 1 time og 16 minutter - 1 time og 30 minutter<br>◯ 1time og 31 minutter - 1 time og 45 minutter<br>◯ 1time og 46 minutter - 2 timer<br>◯ Mere end 2 timer

Hvilke fysiske aktiviteter har du lavet i din 10´er pause i skolen i dag?

- ☐ Ingen
- ☐ Aerobics, Dans
- ☐ Atletik
- ☐ Badminton, Tennis, Squash, Bordtennis
- ☐ Beachvolley
- ☐ Cykling, Spinning, Kondicykel
- ☐ E-sport
- ☐ Floorball
- ☐ Fodbold
- ☐ Gemmeleg, Firkant, Spark til dåsen, Fangeleg/Tik
- ☐ Golf
- ☐ Gymnastik, Rytme, Spring, Tumbling
- ☐ Gåtur
- ☐ Håndbold, Basketball, Hockey
- ☐ Høvdingebold, Rundbold
- ☐ Is-skøjteløb, Ishockey
- ☐ Kampsport (f.eks. karate, boksning, taekwondo)
- ☐ Løb
- ☐ Parkour
- ☐ Rugby, Amerikansk fodbold
- ☐ Skateboard, Løbehjul, Rulleskøjter
- ☐ Styrketræning
- ☐ Svømning
- ☐ Volleyball
- ☐ Andet

Hvor lang tid har du brugt på AEROBICS, DANS i skolen i 10´er pausen?

- ◯ 0-15 minutter
- ◯ 16-30 minutter
- ◯ 31-45 minutter
- ◯ 46-59 minutter
- ◯ 1 time - 1 time og 15 minutter
- ◯ 1 time og 16 minutter - 1 time og 30 minutter
- ◯ 1time og 31 minutter - 1 time og 45 minutter
- ◯ 1time og 46 minutter - 2 timer
- ◯ Mere end 2 timer

Hvor lang tid har du brugt på ATLETIK i skolen i 10´er pausen?

- ◯ 0-15 minutter
- ◯ 16-30 minutter
- ◯ 31-45 minutter
- ◯ 46-59 minutter
- ◯ 1 time - 1 time og 15 minutter
- ◯ 1 time og 16 minutter - 1 time og 30 minutter
- ◯ 1time og 31 minutter - 1 time og 45 minutter
- ◯ 1time og 46 minutter - 2 timer
- ◯ Mere end 2 timer

Hvor lang tid har du brugt på BADMINTON, TENNIS, SQUASH, BORDTENNIS i skolen i 10´er pausen?

- ◯ 0-15 minutter
- ◯ 16-30 minutter
- ◯ 31-45 minutter
- ◯ 46-59 minutter
- ◯ 1 time - 1 time og 15 minutter
- ◯ 1 time og 16 minutter - 1 time og 30 minutter
- ◯ 1time og 31 minutter - 1 time og 45 minutter
- ◯ 1time og 46 minutter - 2 timer
- ◯ Mere end 2 timer

Hvor lang tid har du brugt på BEACHVOLLEY i skolen i 10´er pausen?	◯ 0-15 minutter<br>◯ 16-30 minutter<br>◯ 31-45 minutter<br>◯ 46-59 minutter<br>◯ 1 time - 1 time og 15 minutter<br>◯ 1 time og 16 minutter - 1 time og 30 minutter<br>◯ 1time og 31 minutter - 1 time og 45 minutter<br>◯ 1time og 46 minutter - 2 timer<br>◯ Mere end 2 timer
Hvor lang tid har du brugt på CYKLING, SPINNING, KONDICYKEL i skolen i 10´er pausen?	◯ 0-15 minutter<br>◯ 16-30 minutter<br>◯ 31-45 minutter<br>◯ 46-59 minutter<br>◯ 1 time - 1 time og 15 minutter<br>◯ 1 time og 16 minutter - 1 time og 30 minutter<br>◯ 1time og 31 minutter - 1 time og 45 minutter<br>◯ 1time og 46 minutter - 2 timer<br>◯ Mere end 2 timer
Hvor lang tid har du brugt på E-SPORT i skolen i 10´er pausen?	◯ 0-15 minutter<br>◯ 16-30 minutter<br>◯ 31-45 minutter<br>◯ 46-59 minutter<br>◯ 1 time - 1 time og 15 minutter<br>◯ 1 time og 16 minutter - 1 time og 30 minutter<br>◯ 1time og 31 minutter - 1 time og 45 minutter<br>◯ 1time og 46 minutter - 2 timer<br>◯ Mere end 2 timer
Hvor lang tid har du brugt på FLOORBALL i skolen i 10´er pausen?	◯ 0-15 minutter<br>◯ 16-30 minutter<br>◯ 31-45 minutter<br>◯ 46-59 minutter<br>◯ 1 time - 1 time og 15 minutter<br>◯ 1 time og 16 minutter - 1 time og 30 minutter<br>◯ 1time og 31 minutter - 1 time og 45 minutter<br>◯ 1time og 46 minutter - 2 timer<br>◯ Mere end 2 timer
Hvor lang tid har du brugt på FODBOLD i skolen i 10´er pausen?	◯ 0-15 minutter<br>◯ 16-30 minutter<br>◯ 31-45 minutter<br>◯ 46-59 minutter<br>◯ 1 time - 1 time og 15 minutter<br>◯ 1 time og 16 minutter - 1 time og 30 minutter<br>◯ 1time og 31 minutter - 1 time og 45 minutter<br>◯ 1time og 46 minutter - 2 timer<br>◯ Mere end 2 timer
Hvor lang tid har du brugt på GEMMELEG, FIRKANT, SPARK TIL DÅSEN, FANGELEG/TIK, i skolen i 10´er pausen?	◯ 0-15 minutter<br>◯ 16-30 minutter<br>◯ 31-45 minutter<br>◯ 46-59 minutter<br>◯ 1 time - 1 time og 15 minutter<br>◯ 1 time og 16 minutter - 1 time og 30 minutter<br>◯ 1time og 31 minutter - 1 time og 45 minutter<br>◯ 1time og 46 minutter - 2 timer<br>◯ Mere end 2 timer

Hvor lang tid har du brugt på GOLF i skolen i 10´er pausen?	◯ 0-15 minutter<br>◯ 16-30 minutter<br>◯ 31-45 minutter<br>◯ 46-59 minutter<br>◯ 1 time - 1 time og 15 minutter<br>◯ 1 time og 16 minutter - 1 time og 30 minutter<br>◯ 1time og 31 minutter - 1 time og 45 minutter<br>◯ 1time og 46 minutter - 2 timer<br>◯ Mere end 2 timer
Hvor lang tid har du brugt på GYMNASTIK i skolen i 10´er pausen?	◯ 0-15 minutter<br>◯ 16-30 minutter<br>◯ 31-45 minutter<br>◯ 46-59 minutter<br>◯ 1 time - 1 time og 15 minutter<br>◯ 1 time og 16 minutter - 1 time og 30 minutter<br>◯ 1time og 31 minutter - 1 time og 45 minutter<br>◯ 1time og 46 minutter - 2 timer<br>◯ Mere end 2 timer
Hvor lang tid har du brugt på GÅTUR i skolen i 10´er pausen?	◯ 0-15 minutter<br>◯ 16-30 minutter<br>◯ 31-45 minutter<br>◯ 46-59 minutter<br>◯ 1 time - 1 time og 15 minutter<br>◯ 1 time og 16 minutter - 1 time og 30 minutter<br>◯ 1time og 31 minutter - 1 time og 45 minutter<br>◯ 1time og 46 minutter - 2 timer<br>◯ Mere end 2 timer
Hvor lang tid har du brugt på HÅNDBOLD, BASKETBALL, HOCKEY i skolen i 10´er pausen?	◯ 0-15 minutter<br>◯ 16-30 minutter<br>◯ 31-45 minutter<br>◯ 46-59 minutter<br>◯ 1 time - 1 time og 15 minutter<br>◯ 1 time og 16 minutter - 1 time og 30 minutter<br>◯ 1time og 31 minutter - 1 time og 45 minutter<br>◯ 1time og 46 minutter - 2 timer<br>◯ Mere end 2 timer
Hvor lang tid har du brugt på HØVDINGEBOLD, RUNDBOLD i skolen i 10´er pausen?	◯ 0-15 minutter<br>◯ 16-30 minutter<br>◯ 31-45 minutter<br>◯ 46-59 minutter<br>◯ 1 time - 1 time og 15 minutter<br>◯ 1 time og 16 minutter - 1 time og 30 minutter<br>◯ 1time og 31 minutter - 1 time og 45 minutter<br>◯ 1time og 46 minutter - 2 timer<br>◯ Mere end 2 timer
Hvor lang tid har du brugt på IS-SKØJTELØB, ISHOCKEY i skolen i 10´er pausen?	◯ 0-15 minutter<br>◯ 16-30 minutter<br>◯ 31-45 minutter<br>◯ 46-59 minutter<br>◯ 1 time - 1 time og 15 minutter<br>◯ 1 time og 16 minutter - 1 time og 30 minutter<br>◯ 1time og 31 minutter - 1 time og 45 minutter<br>◯ 1time og 46 minutter - 2 timer<br>◯ Mere end 2 timer

Hvor lang tid har du brugt på KAMPSPORT (f.eks. karate, boksning, teakwondo) i skolen i 10´er pausen?	◯ 0-15 minutter<br>◯ 16-30 minutter<br>◯ 31-45 minutter<br>◯ 46-59 minutter<br>◯ 1 time - 1 time og 15 minutter<br>◯ 1 time og 16 minutter - 1 time og 30 minutter<br>◯ 1time og 31 minutter - 1 time og 45 minutter<br>◯ 1time og 46 minutter - 2 timer<br>◯ Mere end 2 timer
Hvor lang tid har du brugt på LØB i skolen i 10´er pausen?	◯ 0-15 minutter<br>◯ 16-30 minutter<br>◯ 31-45 minutter<br>◯ 46-59 minutter<br>◯ 1 time - 1 time og 15 minutter<br>◯ 1 time og 16 minutter - 1 time og 30 minutter<br>◯ 1time og 31 minutter - 1 time og 45 minutter<br>◯ 1time og 46 minutter - 2 timer<br>◯ Mere end 2 timer
Hvor lang tid har du brugt på PARKOUR i skolen i 10´er pausen?	◯ 0-15 minutter<br>◯ 16-30 minutter<br>◯ 31-45 minutter<br>◯ 46-59 minutter<br>◯ 1 time - 1 time og 15 minutter<br>◯ 1 time og 16 minutter - 1 time og 30 minutter<br>◯ 1time og 31 minutter - 1 time og 45 minutter<br>◯ 1time og 46 minutter - 2 timer<br>◯ Mere end 2 timer
Hvor lang tid har du brugt på Rugby, Amerikansk fodbold i skolen i 10´er pausen?	◯ 0-15 minutter<br>◯ 16-30 minutter<br>◯ 31-45 minutter<br>◯ 46-59 minutter<br>◯ 1 time - 1 time og 15 minutter<br>◯ 1 time og 16 minutter - 1 time og 30 minutter<br>◯ 1time og 31 minutter - 1 time og 45 minutter<br>◯ 1time og 46 minutter - 2 timer<br>◯ Mere end 2 timer
Hvor lang tid har du brugt på SKATEBOARD, LØBEHJUL, RULLESKØJTER i skolen i 10´er pausen?	◯ 0-15 minutter<br>◯ 16-30 minutter<br>◯ 31-45 minutter<br>◯ 46-59 minutter<br>◯ 1 time - 1 time og 15 minutter<br>◯ 1 time og 16 minutter - 1 time og 30 minutter<br>◯ 1time og 31 minutter - 1 time og 45 minutter<br>◯ 1time og 46 minutter - 2 timer<br>◯ Mere end 2 timer
Hvor lang tid har du brugt på STYRKETRÆNING i skolen i 10´er pausen?	◯ 0-15 minutter<br>◯ 16-30 minutter<br>◯ 31-45 minutter<br>◯ 46-59 minutter<br>◯ 1 time - 1 time og 15 minutter<br>◯ 1 time og 16 minutter - 1 time og 30 minutter<br>◯ 1time og 31 minutter - 1 time og 45 minutter<br>◯ 1time og 46 minutter - 2 timer<br>◯ Mere end 2 timer

Hvor lang tid har du brugt på SVØMNING i skolen i 10´er pausen?	◯ 0-15 minutter<br>◯ 16-30 minutter<br>◯ 31-45 minutter<br>◯ 46-59 minutter<br>◯ 1 time - 1 time og 15 minutter<br>◯ 1 time og 16 minutter - 1 time og 30 minutter<br>◯ 1time og 31 minutter - 1 time og 45 minutter<br>◯ 1time og 46 minutter - 2 timer<br>◯ Mere end 2 timer
Hvor lang tid har du brugt på VOLLEYBALL i skolen i 10´er pausen?	◯ 0-15 minutter<br>◯ 16-30 minutter<br>◯ 31-45 minutter<br>◯ 46-59 minutter<br>◯ 1 time - 1 time og 15 minutter<br>◯ 1 time og 16 minutter - 1 time og 30 minutter<br>◯ 1time og 31 minutter - 1 time og 45 minutter<br>◯ 1time og 46 minutter - 2 timer<br>◯ Mere end 2 timer
Hvilken anden fysisk aktivitet har du lavet?	__________________________________
Hvor lang tid har du brugt på denne aktivitet?	◯ 0-15 minutter<br>◯ 16-30 minutter<br>◯ 31-45 minutter<br>◯ 46-59 minutter<br>◯ 1 time - 1 time og 15 minutter<br>◯ 1 time og 16 minutter - 1 time og 30 minutter<br>◯ 1time og 31 minutter - 1 time og 45 minutter<br>◯ 1time og 46 minutter - 2 timer<br>◯ Mere end 2 timer
Hvilke fysiske aktiviteter har du lavet i timerne efter din 10´er pause og før 12´er pause i dag?	☐ Ingen<br>☐ Aerobics, Dans<br>☐ Atletik<br>☐ Badminton, Tennis, Squash, Bordtennis<br>☐ Beachvolley<br>☐ Cykling, Spinning, Kondicykel<br>☐ E-sport<br>☐ Floorball<br>☐ Fodbold<br>☐ Gemmeleg, Firkant, Spark til dåsen, Fangeleg/Tik<br>☐ Golf<br>☐ Gymnastik, Rytme, Spring, Tumbling<br>☐ Gåtur<br>☐ Håndbold, Basketball, Hockey<br>☐ Høvdingebold, Rundbold<br>☐ Is-skøjteløb, Ishockey<br>☐ Kampsport (f.eks. karate, boksning, taekwondo)<br>☐ Løb<br>☐ Parkour<br>☐ Rugby, Amerikansk fodbold<br>☐ Skateboard, Løbehjul, Rulleskøjter<br>☐ Styrketræning<br>☐ Svømning<br>☐ Volleyball<br>☐ Andet

Hvor lang tid har du brugt på AEROBICS, DANS i skolen før 12´er pausen?	◯ 0-15 minutter<br>◯ 16-30 minutter<br>◯ 31-45 minutter<br>◯ 46-59 minutter<br>◯ 1 time - 1 time og 15 minutter<br>◯ 1 time og 16 minutter - 1 time og 30 minutter<br>◯ 1time og 31 minutter - 1 time og 45 minutter<br>◯ 1time og 46 minutter - 2 timer<br>◯ Mere end 2 timer
Hvor lang tid har du brugt på ATLETIK i skolen før 12´er pausen?	◯ 0-15 minutter<br>◯ 16-30 minutter<br>◯ 31-45 minutter<br>◯ 46-59 minutter<br>◯ 1 time - 1 time og 15 minutter<br>◯ 1 time og 16 minutter - 1 time og 30 minutter<br>◯ 1time og 31 minutter - 1 time og 45 minutter<br>◯ 1time og 46 minutter - 2 timer<br>◯ Mere end 2 timer
Hvor lang tid har du brugt på BADMINTON, TENNIS, SQUASH, BORDTENNIS i skolen før 10´er pausen?	◯ 0-15 minutter<br>◯ 16-30 minutter<br>◯ 31-45 minutter<br>◯ 46-59 minutter<br>◯ 1 time - 1 time og 15 minutter<br>◯ 1 time og 16 minutter - 1 time og 30 minutter<br>◯ 1time og 31 minutter - 1 time og 45 minutter<br>◯ 1time og 46 minutter - 2 timer<br>◯ Mere end 2 timer
Hvor lang tid har du brugt på BEACHVOLLEY i skolen før 12´er pausen?	◯ 0-15 minutter<br>◯ 16-30 minutter<br>◯ 31-45 minutter<br>◯ 46-59 minutter<br>◯ 1 time - 1 time og 15 minutter<br>◯ 1 time og 16 minutter - 1 time og 30 minutter<br>◯ 1time og 31 minutter - 1 time og 45 minutter<br>◯ 1time og 46 minutter - 2 timer<br>◯ Mere end 2 timer
Hvor lang tid har du brugt på CYKLING, SPINNING, KONDICYKEL i skolen før 12´er pausen?	◯ 0-15 minutter<br>◯ 16-30 minutter<br>◯ 31-45 minutter<br>◯ 46-59 minutter<br>◯ 1 time - 1 time og 15 minutter<br>◯ 1 time og 16 minutter - 1 time og 30 minutter<br>◯ 1time og 31 minutter - 1 time og 45 minutter<br>◯ 1time og 46 minutter - 2 timer<br>◯ Mere end 2 timer
Hvor lang tid har du brugt på E-SPORT i skolen før 12´er pausen?	◯ 0-15 minutter<br>◯ 16-30 minutter<br>◯ 31-45 minutter<br>◯ 46-59 minutter<br>◯ 1 time - 1 time og 15 minutter<br>◯ 1 time og 16 minutter - 1 time og 30 minutter<br>◯ 1time og 31 minutter - 1 time og 45 minutter<br>◯ 1time og 46 minutter - 2 timer<br>◯ Mere end 2 timer

Hvor lang tid har du brugt på FLOORBALL i skolen før 12´er pausen?	◯ 0-15 minutter<br>◯ 16-30 minutter<br>◯ 31-45 minutter<br>◯ 46-59 minutter<br>◯ 1 time - 1 time og 15 minutter<br>◯ 1 time og 16 minutter - 1 time og 30 minutter<br>◯ 1time og 31 minutter - 1 time og 45 minutter<br>◯ 1time og 46 minutter - 2 timer<br>◯ Mere end 2 timer
Hvor lang tid har du brugt på FODBOLD i skolen før 12´er pausen?	◯ 0-15 minutter<br>◯ 16-30 minutter<br>◯ 31-45 minutter<br>◯ 46-59 minutter<br>◯ 1 time - 1 time og 15 minutter<br>◯ 1 time og 16 minutter - 1 time og 30 minutter<br>◯ 1time og 31 minutter - 1 time og 45 minutter<br>◯ 1time og 46 minutter - 2 timer<br>◯ Mere end 2 timer
Hvor lang tid har du brugt på GEMMELEG, FIRKANT, SPARK TIL DÅSEN, FANGELEG/TIK, i skolen før 10´er pausen?	◯ 0-15 minutter<br>◯ 16-30 minutter<br>◯ 31-45 minutter<br>◯ 46-59 minutter<br>◯ 1 time - 1 time og 15 minutter<br>◯ 1 time og 16 minutter - 1 time og 30 minutter<br>◯ 1time og 31 minutter - 1 time og 45 minutter<br>◯ 1time og 46 minutter - 2 timer<br>◯ Mere end 2 timer
Hvor lang tid har du brugt på GOLF i skolen før 12´er pausen?	◯ 0-15 minutter<br>◯ 16-30 minutter<br>◯ 31-45 minutter<br>◯ 46-59 minutter<br>◯ 1 time - 1 time og 15 minutter<br>◯ 1 time og 16 minutter - 1 time og 30 minutter<br>◯ 1time og 31 minutter - 1 time og 45 minutter<br>◯ 1time og 46 minutter - 2 timer<br>◯ Mere end 2 timer
Hvor lang tid har du brugt på GYMNASTIK i skolen før 12´er pausen?	◯ 0-15 minutter<br>◯ 16-30 minutter<br>◯ 31-45 minutter<br>◯ 46-59 minutter<br>◯ 1 time - 1 time og 15 minutter<br>◯ 1 time og 16 minutter - 1 time og 30 minutter<br>◯ 1time og 31 minutter - 1 time og 45 minutter<br>◯ 1time og 46 minutter - 2 timer<br>◯ Mere end 2 timer
Hvor lang tid har du brugt på GÅTUR i skolen før 12´er pausen?	◯ 0-15 minutter<br>◯ 16-30 minutter<br>◯ 31-45 minutter<br>◯ 46-59 minutter<br>◯ 1 time - 1 time og 15 minutter<br>◯ 1 time og 16 minutter - 1 time og 30 minutter<br>◯ 1time og 31 minutter - 1 time og 45 minutter<br>◯ 1time og 46 minutter - 2 timer<br>◯ Mere end 2 timer

Hvor lang tid har du brugt på HÅNDBOLD, BASKETBALL, HOCKEY i skolen før 12´er pausen?	◯ 0-15 minutter<br>◯ 16-30 minutter<br>◯ 31-45 minutter<br>◯ 46-59 minutter<br>◯ 1 time - 1 time og 15 minutter<br>◯ 1 time og 16 minutter - 1 time og 30 minutter<br>◯ 1time og 31 minutter - 1 time og 45 minutter<br>◯ 1time og 46 minutter - 2 timer<br>◯ Mere end 2 timer
Hvor lang tid har du brugt på HØVDINGEBOLD, RUNDBOLD i skolen før 12´er pausen?	◯ 0-15 minutter<br>◯ 16-30 minutter<br>◯ 31-45 minutter<br>◯ 46-59 minutter<br>◯ 1 time - 1 time og 15 minutter<br>◯ 1 time og 16 minutter - 1 time og 30 minutter<br>◯ 1time og 31 minutter - 1 time og 45 minutter<br>◯ 1time og 46 minutter - 2 timer<br>◯ Mere end 2 timer
Hvor lang tid har du brugt på IS-SKØJTELØB, ISHOCKEY i skolen før 12´er pausen?	◯ 0-15 minutter<br>◯ 16-30 minutter<br>◯ 31-45 minutter<br>◯ 46-59 minutter<br>◯ 1 time - 1 time og 15 minutter<br>◯ 1 time og 16 minutter - 1 time og 30 minutter<br>◯ 1time og 31 minutter - 1 time og 45 minutter<br>◯ 1time og 46 minutter - 2 timer<br>◯ Mere end 2 timer
Hvor lang tid har du brugt på KAMPSPORT (f.eks. karate, boksning, Teakwondo) i skolen før 12´er pausen?	◯ 0-15 minutter<br>◯ 16-30 minutter<br>◯ 31-45 minutter<br>◯ 46-59 minutter<br>◯ 1 time - 1 time og 15 minutter<br>◯ 1 time og 16 minutter - 1 time og 30 minutter<br>◯ 1time og 31 minutter - 1 time og 45 minutter<br>◯ 1time og 46 minutter - 2 timer<br>◯ Mere end 2 timer
Hvor lang tid har du brugt på LØB i skolen før 12´er pausen?	◯ 0-15 minutter<br>◯ 16-30 minutter<br>◯ 31-45 minutter<br>◯ 46-59 minutter<br>◯ 1 time - 1 time og 15 minutter<br>◯ 1 time og 16 minutter - 1 time og 30 minutter<br>◯ 1time og 31 minutter - 1 time og 45 minutter<br>◯ 1time og 46 minutter - 2 timer<br>◯ Mere end 2 timer
Hvor lang tid har du brugt på PARKOUR i skolen før 12´er pausen?	◯ 0-15 minutter<br>◯ 16-30 minutter<br>◯ 31-45 minutter<br>◯ 46-59 minutter<br>◯ 1 time - 1 time og 15 minutter<br>◯ 1 time og 16 minutter - 1 time og 30 minutter<br>◯ 1time og 31 minutter - 1 time og 45 minutter<br>◯ 1time og 46 minutter - 2 timer<br>◯ Mere end 2 timer

Hvor lang tid har du brugt på Rugby, Amerikansk fodbold i skolen før 12´er pausen?	◯ 0-15 minutter<br>◯ 16-30 minutter<br>◯ 31-45 minutter<br>◯ 46-59 minutter<br>◯ 1 time - 1 time og 15 minutter<br>◯ 1 time og 16 minutter - 1 time og 30 minutter<br>◯ 1time og 31 minutter - 1 time og 45 minutter<br>◯ 1time og 46 minutter - 2 timer<br>◯ Mere end 2 timer
Hvor lang tid har du brugt på SKATEBOARD, LØBEHJUL, RULLESKØJTER i skolen før 12´er pausen?	◯ 0-15 minutter<br>◯ 16-30 minutter<br>◯ 31-45 minutter<br>◯ 46-59 minutter<br>◯ 1 time - 1 time og 15 minutter<br>◯ 1 time og 16 minutter - 1 time og 30 minutter<br>◯ 1time og 31 minutter - 1 time og 45 minutter<br>◯ 1time og 46 minutter - 2 timer<br>◯ Mere end 2 timer
Hvor lang tid har du brugt på STYRKETRÆNING i skolen før 12´er pausen?	◯ 0-15 minutter<br>◯ 16-30 minutter<br>◯ 31-45 minutter<br>◯ 46-59 minutter<br>◯ 1 time - 1 time og 15 minutter<br>◯ 1 time og 16 minutter - 1 time og 30 minutter<br>◯ 1time og 31 minutter - 1 time og 45 minutter<br>◯ 1time og 46 minutter - 2 timer<br>◯ Mere end 2 timer
Hvor lang tid har du brugt på SVØMNING i skolen før 12´er pausen?	◯ 0-15 minutter<br>◯ 16-30 minutter<br>◯ 31-45 minutter<br>◯ 46-59 minutter<br>◯ 1 time - 1 time og 15 minutter<br>◯ 1 time og 16 minutter - 1 time og 30 minutter<br>◯ 1time og 31 minutter - 1 time og 45 minutter<br>◯ 1time og 46 minutter - 2 timer<br>◯ Mere end 2 timer
Hvor lang tid har du brugt på VOLLEYBALL i skolen før 12´er pausen?	◯ 0-15 minutter<br>◯ 16-30 minutter<br>◯ 31-45 minutter<br>◯ 46-59 minutter<br>◯ 1 time - 1 time og 15 minutter<br>◯ 1 time og 16 minutter - 1 time og 30 minutter<br>◯ 1time og 31 minutter - 1 time og 45 minutter<br>◯ 1time og 46 minutter - 2 timer<br>◯ Mere end 2 timer
Hvilken anden fysisk aktivitet har du lavet?	__________________________

Hvor lang tid har du brugt på denne aktivitet?	◯ 0-15 minutter<br>◯ 16-30 minutter<br>◯ 31-45 minutter<br>◯ 46-59 minutter<br>◯ 1 time - 1 time og 15 minutter<br>◯ 1 time og 16 minutter - 1 time og 30 minutter<br>◯ 1time og 31 minutter - 1 time og 45 minutter<br>◯ 1time og 46 minutter - 2 timer<br>◯ Mere end 2 timer
Hvilke aktiviteter har du lavet i din 12´er pause i skolen i dag?	☐ Ingen<br>☐ Aerobics, Dans<br>☐ Atletik<br>☐ Badminton, Tennis, Squash, Bordtennis<br>☐ Beachvolley<br>☐ Cykling, Spinning, Kondicykel<br>☐ E-sport, computerspil<br>☐ Floorball<br>☐ Fodbold<br>☐ Gemmeleg, Firkant, Spark til dåsen, Fangeleg/Tik<br>☐ Golf<br>☐ Gymnastik, Rytme, Spring, Tumbling<br>☐ Gåtur<br>☐ Håndbold, Basketball, Hockey<br>☐ Høvdingebold, Rundbold<br>☐ Is-skøjteløb, Ishockey<br>☐ Kampsport (f.eks. karate, boksning, taekwondo)<br>☐ Løb<br>☐ Parkour<br>☐ Rugby, Amerikansk fodbold<br>☐ Skateboard, Løbehjul, Rulleskøjter<br>☐ Styrketræning<br>☐ Svømning<br>☐ Volleyball<br>☐ Andet
Hvor lang tid har du brugt på AEROBICS, DANS i skolen i 12´er pausen?	◯ 0-15 minutter<br>◯ 16-30 minutter<br>◯ 31-45 minutter<br>◯ 46-59 minutter<br>◯ 1 time - 1 time og 15 minutter<br>◯ 1 time og 16 minutter - 1 time og 30 minutter<br>◯ 1time og 31 minutter - 1 time og 45 minutter<br>◯ 1time og 46 minutter - 2 timer<br>◯ Mere end 2 timer
Hvor lang tid har du brugt på ATLETIK i skolen i 12´er pausen?	◯ 0-15 minutter<br>◯ 16-30 minutter<br>◯ 31-45 minutter<br>◯ 46-59 minutter<br>◯ 1 time - 1 time og 15 minutter<br>◯ 1 time og 16 minutter - 1 time og 30 minutter<br>◯ 1time og 31 minutter - 1 time og 45 minutter<br>◯ 1time og 46 minutter - 2 timer<br>◯ Mere end 2 timer

Hvor lang tid har du brugt på BADMINTON, TENNIS, SQUASH, BORDTENNIS i skolen i 12´er pausen?	◯ 0-15 minutter<br>◯ 16-30 minutter<br>◯ 31-45 minutter<br>◯ 46-59 minutter<br>◯ 1 time - 1 time og 15 minutter<br>◯ 1 time og 16 minutter - 1 time og 30 minutter<br>◯ 1time og 31 minutter - 1 time og 45 minutter<br>◯ 1time og 46 minutter - 2 timer<br>◯ Mere end 2 timer
Hvor lang tid har du brugt på BEACHVOLLEY i skolen i 12´er pausen?	◯ 0-15 minutter<br>◯ 16-30 minutter<br>◯ 31-45 minutter<br>◯ 46-59 minutter<br>◯ 1 time - 1 time og 15 minutter<br>◯ 1 time og 16 minutter - 1 time og 30 minutter<br>◯ 1time og 31 minutter - 1 time og 45 minutter<br>◯ 1time og 46 minutter - 2 timer<br>◯ Mere end 2 timer
Hvor lang tid har du brugt på CYKLING, SPINNING, KONDICYKEL i skolen i 12´er pausen?	◯ 0-15 minutter<br>◯ 16-30 minutter<br>◯ 31-45 minutter<br>◯ 46-59 minutter<br>◯ 1 time - 1 time og 15 minutter<br>◯ 1 time og 16 minutter - 1 time og 30 minutter<br>◯ 1time og 31 minutter - 1 time og 45 minutter<br>◯ 1time og 46 minutter - 2 timer<br>◯ Mere end 2 timer
Hvor lang tid har du brugt på E-SPORT i skolen i 12´er pausen?	◯ 0-15 minutter<br>◯ 16-30 minutter<br>◯ 31-45 minutter<br>◯ 46-59 minutter<br>◯ 1 time - 1 time og 15 minutter<br>◯ 1 time og 16 minutter - 1 time og 30 minutter<br>◯ 1time og 31 minutter - 1 time og 45 minutter<br>◯ 1time og 46 minutter - 2 timer<br>◯ Mere end 2 timer
Hvor lang tid har du brugt på FLOORBALL i skolen i 12´er pausen?	◯ 0-15 minutter<br>◯ 16-30 minutter<br>◯ 31-45 minutter<br>◯ 46-59 minutter<br>◯ 1 time - 1 time og 15 minutter<br>◯ 1 time og 16 minutter - 1 time og 30 minutter<br>◯ 1time og 31 minutter - 1 time og 45 minutter<br>◯ 1time og 46 minutter - 2 timer<br>◯ Mere end 2 timer
Hvor lang tid har du brugt på FODBOLD i skolen i 12´er pausen?	◯ 0-15 minutter<br>◯ 16-30 minutter<br>◯ 31-45 minutter<br>◯ 46-59 minutter<br>◯ 1 time - 1 time og 15 minutter<br>◯ 1 time og 16 minutter - 1 time og 30 minutter<br>◯ 1time og 31 minutter - 1 time og 45 minutter<br>◯ 1time og 46 minutter - 2 timer<br>◯ Mere end 2 timer

Hvor lang tid har du brugt på GEMMELEG, FIRKANT, SPARK TIL DÅSEN, FANGELEG/TIK, i skolen i 12´er pausen?	◯ 0-15 minutter<br>◯ 16-30 minutter<br>◯ 31-45 minutter<br>◯ 46-59 minutter<br>◯ 1 time - 1 time og 15 minutter<br>◯ 1 time og 16 minutter - 1 time og 30 minutter<br>◯ 1time og 31 minutter - 1 time og 45 minutter<br>◯ 1time og 46 minutter - 2 timer<br>◯ Mere end 2 timer
Hvor lang tid har du brugt på GOLF i skolen i 12´er pausen?	◯ 0-15 minutter<br>◯ 16-30 minutter<br>◯ 31-45 minutter<br>◯ 46-59 minutter<br>◯ 1 time - 1 time og 15 minutter<br>◯ 1 time og 16 minutter - 1 time og 30 minutter<br>◯ 1time og 31 minutter - 1 time og 45 minutter<br>◯ 1time og 46 minutter - 2 timer<br>◯ Mere end 2 timer
Hvor lang tid har du brugt på GYMNASTIK i skolen i 12´er pausen?	◯ 0-15 minutter<br>◯ 16-30 minutter<br>◯ 31-45 minutter<br>◯ 46-59 minutter<br>◯ 1 time - 1 time og 15 minutter<br>◯ 1 time og 16 minutter - 1 time og 30 minutter<br>◯ 1time og 31 minutter - 1 time og 45 minutter<br>◯ 1time og 46 minutter - 2 timer<br>◯ Mere end 2 timer
Hvor lang tid har du brugt på GÅTUR i skolen i 12´er pausen?	◯ 0-15 minutter<br>◯ 16-30 minutter<br>◯ 31-45 minutter<br>◯ 46-59 minutter<br>◯ 1 time - 1 time og 15 minutter<br>◯ 1 time og 16 minutter - 1 time og 30 minutter<br>◯ 1time og 31 minutter - 1 time og 45 minutter<br>◯ 1time og 46 minutter - 2 timer<br>◯ Mere end 2 timer
Hvor lang tid har du brugt på HÅNDBOLD, BASKETBALL, HOCKEY i skolen i 12´er pausen?	◯ 0-15 minutter<br>◯ 16-30 minutter<br>◯ 31-45 minutter<br>◯ 46-59 minutter<br>◯ 1 time - 1 time og 15 minutter<br>◯ 1 time og 16 minutter - 1 time og 30 minutter<br>◯ 1time og 31 minutter - 1 time og 45 minutter<br>◯ 1time og 46 minutter - 2 timer<br>◯ Mere end 2 timer
Hvor lang tid har du brugt på HØVDINGEBOLD, RUNDBOLD i skolen i 12´er pausen?	◯ 0-15 minutter<br>◯ 16-30 minutter<br>◯ 31-45 minutter<br>◯ 46-59 minutter<br>◯ 1 time - 1 time og 15 minutter<br>◯ 1 time og 16 minutter - 1 time og 30 minutter<br>◯ 1time og 31 minutter - 1 time og 45 minutter<br>◯ 1time og 46 minutter - 2 timer<br>◯ Mere end 2 timer

Hvor lang tid har du brugt på IS-SKØJTELØB, ISHOCKEY i skolen i 12´er pausen?	◯ 0-15 minutter<br>◯ 16-30 minutter<br>◯ 31-45 minutter<br>◯ 46-59 minutter<br>◯ 1 time - 1 time og 15 minutter<br>◯ 1 time og 16 minutter - 1 time og 30 minutter<br>◯ 1time og 31 minutter - 1 time og 45 minutter<br>◯ 1time og 46 minutter - 2 timer<br>◯ Mere end 2 timer
Hvor lang tid har du brugt på KAMPSPORT (f.eks. karate, boksning, teakwondo) i skolen i 12´er pausen?	◯ 0-15 minutter<br>◯ 16-30 minutter<br>◯ 31-45 minutter<br>◯ 46-59 minutter<br>◯ 1 time - 1 time og 15 minutter<br>◯ 1 time og 16 minutter - 1 time og 30 minutter<br>◯ 1time og 31 minutter - 1 time og 45 minutter<br>◯ 1time og 46 minutter - 2 timer<br>◯ Mere end 2 timer
Hvor lang tid har du brugt på LØB i skolen i 12´er pausen?	◯ 0-15 minutter<br>◯ 16-30 minutter<br>◯ 31-45 minutter<br>◯ 46-59 minutter<br>◯ 1 time - 1 time og 15 minutter<br>◯ 1 time og 16 minutter - 1 time og 30 minutter<br>◯ 1time og 31 minutter - 1 time og 45 minutter<br>◯ 1time og 46 minutter - 2 timer<br>◯ Mere end 2 timer
Hvor lang tid har du brugt på PARKOUR i skolen i 12´er pausen?	◯ 0-15 minutter<br>◯ 16-30 minutter<br>◯ 31-45 minutter<br>◯ 46-59 minutter<br>◯ 1 time - 1 time og 15 minutter<br>◯ 1 time og 16 minutter - 1 time og 30 minutter<br>◯ 1time og 31 minutter - 1 time og 45 minutter<br>◯ 1time og 46 minutter - 2 timer<br>◯ Mere end 2 timer
Hvor lang tid har du brugt på Rugby, Amerikansk fodbold i skolen i 12´er pausen?	◯ 0-15 minutter<br>◯ 16-30 minutter<br>◯ 31-45 minutter<br>◯ 46-59 minutter<br>◯ 1 time - 1 time og 15 minutter<br>◯ 1 time og 16 minutter - 1 time og 30 minutter<br>◯ 1time og 31 minutter - 1 time og 45 minutter<br>◯ 1time og 46 minutter - 2 timer<br>◯ Mere end 2 timer
Hvor lang tid har du brugt på SKATEBOARD, LØBEHJUL, RULLESKØJTER i skolen i 12´er pausen?	◯ 0-15 minutter<br>◯ 16-30 minutter<br>◯ 31-45 minutter<br>◯ 46-59 minutter<br>◯ 1 time - 1 time og 15 minutter<br>◯ 1 time og 16 minutter - 1 time og 30 minutter<br>◯ 1time og 31 minutter - 1 time og 45 minutter<br>◯ 1time og 46 minutter - 2 timer<br>◯ Mere end 2 timer

Hvor lang tid har du brugt på STYRKETRÆNING i skolen 1 12´er pausen?	◯ 0-15 minutter<br>◯ 16-30 minutter<br>◯ 31-45 minutter<br>◯ 46-59 minutter<br>◯ 1 time - 1 time og 15 minutter<br>◯ 1 time og 16 minutter - 1 time og 30 minutter<br>◯ 1time og 31 minutter - 1 time og 45 minutter<br>◯ 1time og 46 minutter - 2 timer<br>◯ Mere end 2 timer
Hvor lang tid har du brugt på SVØMNING i skolen i 12´er pausen?	◯ 0-15 minutter<br>◯ 16-30 minutter<br>◯ 31-45 minutter<br>◯ 46-59 minutter<br>◯ 1 time - 1 time og 15 minutter<br>◯ 1 time og 16 minutter - 1 time og 30 minutter<br>◯ 1time og 31 minutter - 1 time og 45 minutter<br>◯ 1time og 46 minutter - 2 timer<br>◯ Mere end 2 timer
Hvor lang tid har du brugt på VOLLEYBALL i skolen i 12´er pausen?	◯ 0-15 minutter<br>◯ 16-30 minutter<br>◯ 31-45 minutter<br>◯ 46-59 minutter<br>◯ 1 time - 1 time og 15 minutter<br>◯ 1 time og 16 minutter - 1 time og 30 minutter<br>◯ 1time og 31 minutter - 1 time og 45 minutter<br>◯ 1time og 46 minutter - 2 timer<br>◯ Mere end 2 timer
Hvilken anden aktivitet har du lavet i 12´er pausen?	__________________________________
Hvor lang tid har du brugt på denne aktivitet?	◯ 0-15 minutter<br>◯ 16-30 minutter<br>◯ 31-45 minutter<br>◯ 46-59 minutter<br>◯ 1 time - 1 time og 15 minutter<br>◯ 1 time og 16 minutter - 1 time og 30 minutter<br>◯ 1time og 31 minutter - 1 time og 45 minutter<br>◯ 1time og 46 minutter - 2 timer<br>◯ Mere end 2 timer

Hvilke fysiske aktiviteter har du lavet i timerne efter din 12´er pause i dag?

- ☐ Ingen
- ☐ Aerobics, Dans
- ☐ Atletik
- ☐ Badminton, Tennis, Squash, Bordtennis
- ☐ Beachvolley
- ☐ Cykling, Spinning, Kondicykel
- ☐ E-sport
- ☐ Floorball
- ☐ Fodbold
- ☐ Gemmeleg, Firkant, Spark til dåsen, Fangeleg/Tik
- ☐ Golf
- ☐ Gymnastik, Rytme, Spring, Tumbling
- ☐ Gåtur
- ☐ Håndbold, Basketball, Hockey
- ☐ Høvdingebold, Rundbold
- ☐ Is-skøjteløb, Ishockey
- ☐ Kampsport (f.eks. karate, boksning, taekwondo)
- ☐ Løb
- ☐ Parkour
- ☐ Rugby, Amerikansk fodbold
- ☐ Skateboard, Løbehjul, Rulleskøjter
- ☐ Styrketræning
- ☐ Svømning
- ☐ Volleyball
- ☐ Andet

Hvor lang tid har du brugt på AEROBICS, DANS i skolen efter 12´er pausen?

- ◯ 0-15 minutter
- ◯ 16-30 minutter
- ◯ 31-45 minutter
- ◯ 46-59 minutter
- ◯ 1 time - 1 time og 15 minutter
- ◯ 1 time og 16 minutter - 1 time og 30 minutter
- ◯ 1time og 31 minutter - 1 time og 45 minutter
- ◯ 1time og 46 minutter - 2 timer
- ◯ Mere end 2 timer

Hvor lang tid har du brugt på ATLETIK i skolen efter 12´er pausen?

- ◯ 0-15 minutter
- ◯ 16-30 minutter
- ◯ 31-45 minutter
- ◯ 46-59 minutter
- ◯ 1 time - 1 time og 15 minutter
- ◯ 1 time og 16 minutter - 1 time og 30 minutter
- ◯ 1time og 31 minutter - 1 time og 45 minutter
- ◯ 1time og 46 minutter - 2 timer
- ◯ Mere end 2 timer

Hvor lang tid har du brugt på BADMINTON, TENNIS, SQUASH, BORDTENNIS i skolen efer 12´er pausen?

- ◯ 0-15 minutter
- ◯ 16-30 minutter
- ◯ 31-45 minutter
- ◯ 46-59 minutter
- ◯ 1 time - 1 time og 15 minutter
- ◯ 1 time og 16 minutter - 1 time og 30 minutter
- ◯ 1time og 31 minutter - 1 time og 45 minutter
- ◯ 1time og 46 minutter - 2 timer
- ◯ Mere end 2 timer

Hvor lang tid har du brugt på BEACHVOLLEY i skolen efter 12´er pausen?	◯ 0-15 minutter<br>◯ 16-30 minutter<br>◯ 31-45 minutter<br>◯ 46-59 minutter<br>◯ 1 time - 1 time og 15 minutter<br>◯ 1 time og 16 minutter - 1 time og 30 minutter<br>◯ 1time og 31 minutter - 1 time og 45 minutter<br>◯ 1time og 46 minutter - 2 timer<br>◯ Mere end 2 timer
Hvor lang tid har du brugt på CYKLING, SPINNING, KONDICYKEL i skolen efter 12´er pausen?	◯ 0-15 minutter<br>◯ 16-30 minutter<br>◯ 31-45 minutter<br>◯ 46-59 minutter<br>◯ 1 time - 1 time og 15 minutter<br>◯ 1 time og 16 minutter - 1 time og 30 minutter<br>◯ 1time og 31 minutter - 1 time og 45 minutter<br>◯ 1time og 46 minutter - 2 timer<br>◯ Mere end 2 timer
Hvor lang tid har du brugt på E-SPORT i skolen efter 12´er pausen?	◯ 0-15 minutter<br>◯ 16-30 minutter<br>◯ 31-45 minutter<br>◯ 46-59 minutter<br>◯ 1 time - 1 time og 15 minutter<br>◯ 1 time og 16 minutter - 1 time og 30 minutter<br>◯ 1time og 31 minutter - 1 time og 45 minutter<br>◯ 1time og 46 minutter - 2 timer<br>◯ Mere end 2 timer
Hvor lang tid har du brugt på FLOORBALL i skolen efter 12´er pausen?	◯ 0-15 minutter<br>◯ 16-30 minutter<br>◯ 31-45 minutter<br>◯ 46-59 minutter<br>◯ 1 time - 1 time og 15 minutter<br>◯ 1 time og 16 minutter - 1 time og 30 minutter<br>◯ 1time og 31 minutter - 1 time og 45 minutter<br>◯ 1time og 46 minutter - 2 timer<br>◯ Mere end 2 timer
Hvor lang tid har du brugt på FODBOLD i skolen efter 12´er pausen?	◯ 0-15 minutter<br>◯ 16-30 minutter<br>◯ 31-45 minutter<br>◯ 46-59 minutter<br>◯ 1 time - 1 time og 15 minutter<br>◯ 1 time og 16 minutter - 1 time og 30 minutter<br>◯ 1time og 31 minutter - 1 time og 45 minutter<br>◯ 1time og 46 minutter - 2 timer<br>◯ Mere end 2 timer
Hvor lang tid har du brugt på GEMMELEG, FIRKANT, SPARK TIL DÅSEN, FANGELEG/TIK, i skolen efter 12´er pausen?	◯ 0-15 minutter<br>◯ 16-30 minutter<br>◯ 31-45 minutter<br>◯ 46-59 minutter<br>◯ 1 time - 1 time og 15 minutter<br>◯ 1 time og 16 minutter - 1 time og 30 minutter<br>◯ 1time og 31 minutter - 1 time og 45 minutter<br>◯ 1time og 46 minutter - 2 timer<br>◯ Mere end 2 timer

Hvor lang tid har du brugt på GOLF i skolen efter 12´er pausen?	◯ 0-15 minutter<br>◯ 16-30 minutter<br>◯ 31-45 minutter<br>◯ 46-59 minutter<br>◯ 1 time - 1 time og 15 minutter<br>◯ 1 time og 16 minutter - 1 time og 30 minutter<br>◯ 1time og 31 minutter - 1 time og 45 minutter<br>◯ 1time og 46 minutter - 2 timer<br>◯ Mere end 2 timer
Hvor lang tid har du brugt på GYMNASTIK i skolen efter 12´er pausen?	◯ 0-15 minutter<br>◯ 16-30 minutter<br>◯ 31-45 minutter<br>◯ 46-59 minutter<br>◯ 1 time - 1 time og 15 minutter<br>◯ 1 time og 16 minutter - 1 time og 30 minutter<br>◯ 1time og 31 minutter - 1 time og 45 minutter<br>◯ 1time og 46 minutter - 2 timer<br>◯ Mere end 2 timer
Hvor lang tid har du brugt på GÅTUR i skolen efter 12´er pausen?	◯ 0-15 minutter<br>◯ 16-30 minutter<br>◯ 31-45 minutter<br>◯ 46-59 minutter<br>◯ 1 time - 1 time og 15 minutter<br>◯ 1 time og 16 minutter - 1 time og 30 minutter<br>◯ 1time og 31 minutter - 1 time og 45 minutter<br>◯ 1time og 46 minutter - 2 timer<br>◯ Mere end 2 timer
Hvor lang tid har du brugt på HÅNDBOLD, BASKETBALL, HOCKEY i skolen efter 12´er pausen?	◯ 0-15 minutter<br>◯ 16-30 minutter<br>◯ 31-45 minutter<br>◯ 46-59 minutter<br>◯ 1 time - 1 time og 15 minutter<br>◯ 1 time og 16 minutter - 1 time og 30 minutter<br>◯ 1time og 31 minutter - 1 time og 45 minutter<br>◯ 1time og 46 minutter - 2 timer<br>◯ Mere end 2 timer
Hvor lang tid har du brugt på HØVDINGEBOLD, RUNDBOLD i skolen efter 12´er pausen?	◯ 0-15 minutter<br>◯ 16-30 minutter<br>◯ 31-45 minutter<br>◯ 46-59 minutter<br>◯ 1 time - 1 time og 15 minutter<br>◯ 1 time og 16 minutter - 1 time og 30 minutter<br>◯ 1time og 31 minutter - 1 time og 45 minutter<br>◯ 1time og 46 minutter - 2 timer<br>◯ Mere end 2 timer
Hvor lang tid har du brugt på IS-SKØJTELØB, ISHOCKEY i skolen efter 12´er pausen?	◯ 0-15 minutter<br>◯ 16-30 minutter<br>◯ 31-45 minutter<br>◯ 46-59 minutter<br>◯ 1 time - 1 time og 15 minutter<br>◯ 1 time og 16 minutter - 1 time og 30 minutter<br>◯ 1time og 31 minutter - 1 time og 45 minutter<br>◯ 1time og 46 minutter - 2 timer<br>◯ Mere end 2 timer

Hvor lang tid har du brugt på KAMPSPORT (f.eks. karate, boksning, teakwondo) i skolen efter 12´er pausen?	◯ 0-15 minutter<br>◯ 16-30 minutter<br>◯ 31-45 minutter<br>◯ 46-59 minutter<br>◯ 1 time - 1 time og 15 minutter<br>◯ 1 time og 16 minutter - 1 time og 30 minutter<br>◯ 1time og 31 minutter - 1 time og 45 minutter<br>◯ 1time og 46 minutter - 2 timer<br>◯ Mere end 2 timer
Hvor lang tid har du brugt på LØB i skolen efter 12´er pausen?	◯ 0-15 minutter<br>◯ 16-30 minutter<br>◯ 31-45 minutter<br>◯ 46-59 minutter<br>◯ 1 time - 1 time og 15 minutter<br>◯ 1 time og 16 minutter - 1 time og 30 minutter<br>◯ 1time og 31 minutter - 1 time og 45 minutter<br>◯ 1time og 46 minutter - 2 timer<br>◯ Mere end 2 timer
Hvor lang tid har du brugt på PARKOUR i skolen efter 12´er pausen?	◯ 0-15 minutter<br>◯ 16-30 minutter<br>◯ 31-45 minutter<br>◯ 46-59 minutter<br>◯ 1 time - 1 time og 15 minutter<br>◯ 1 time og 16 minutter - 1 time og 30 minutter<br>◯ 1time og 31 minutter - 1 time og 45 minutter<br>◯ 1time og 46 minutter - 2 timer<br>◯ Mere end 2 timer
Hvor lang tid har du brugt på Rugby, Amerikansk fodbold i skolen efter 12´er pausen?	◯ 0-15 minutter<br>◯ 16-30 minutter<br>◯ 31-45 minutter<br>◯ 46-59 minutter<br>◯ 1 time - 1 time og 15 minutter<br>◯ 1 time og 16 minutter - 1 time og 30 minutter<br>◯ 1time og 31 minutter - 1 time og 45 minutter<br>◯ 1time og 46 minutter - 2 timer<br>◯ Mere end 2 timer
Hvor lang tid har du brugt på SKATEBOARD, LØBEHJUL, RULLESKØJTER i skolen efter 12´er pausen?	◯ 0-15 minutter<br>◯ 16-30 minutter<br>◯ 31-45 minutter<br>◯ 46-59 minutter<br>◯ 1 time - 1 time og 15 minutter<br>◯ 1 time og 16 minutter - 1 time og 30 minutter<br>◯ 1time og 31 minutter - 1 time og 45 minutter<br>◯ 1time og 46 minutter - 2 timer<br>◯ Mere end 2 timer
Hvor lang tid har du brugt på STYRKETRÆNING i skolen efter 12´er pausen?	◯ 0-15 minutter<br>◯ 16-30 minutter<br>◯ 31-45 minutter<br>◯ 46-59 minutter<br>◯ 1 time - 1 time og 15 minutter<br>◯ 1 time og 16 minutter - 1 time og 30 minutter<br>◯ 1time og 31 minutter - 1 time og 45 minutter<br>◯ 1time og 46 minutter - 2 timer<br>◯ Mere end 2 timer

Hvor lang tid har du brugt på SVØMNING i skolen efter 12´er pausen?	◯ 0-15 minutter<br>◯ 16-30 minutter<br>◯ 31-45 minutter<br>◯ 46-59 minutter<br>◯ 1 time - 1 time og 15 minutter<br>◯ 1 time og 16 minutter - 1 time og 30 minutter<br>◯ 1time og 31 minutter - 1 time og 45 minutter<br>◯ 1time og 46 minutter - 2 timer<br>◯ Mere end 2 timer
Hvor lang tid har du brugt på VOLLEYBALL i skolen efter 12´er pausen?	◯ 0-15 minutter<br>◯ 16-30 minutter<br>◯ 31-45 minutter<br>◯ 46-59 minutter<br>◯ 1 time - 1 time og 15 minutter<br>◯ 1 time og 16 minutter - 1 time og 30 minutter<br>◯ 1time og 31 minutter - 1 time og 45 minutter<br>◯ 1time og 46 minutter - 2 timer<br>◯ Mere end 2 timer
Hvilken anden fysisk aktivitet har du lavet?	__________________________________
Hvor lang tid har du brugt på denne aktivitet?	◯ 0-15 minutter<br>◯ 16-30 minutter<br>◯ 31-45 minutter<br>◯ 46-59 minutter<br>◯ 1 time - 1 time og 15 minutter<br>◯ 1 time og 16 minutter - 1 time og 30 minutter<br>◯ 1time og 31 minutter - 1 time og 45 minutter<br>◯ 1time og 46 minutter - 2 timer<br>◯ Mere end 2 timer

Hvilke fysiske aktiviteter har du lavet fra du fik fri fra skole til du skulle spise aftensmad i dag?	☐ Ingen<br>☐ Aerobics, Dans<br>☐ Atletik<br>☐ Badminton, Tennis, Squash, Bordtennis<br>☐ Ballet<br>☐ Beachvolley<br>☐ Cykling, Spinning, Kondicykel<br>☐ E-sport<br>☐ Floorball<br>☐ Fodbold<br>☐ Gemmeleg, Firkant, Spark til dåsen, Fangeleg/Tik<br>☐ Golf<br>☐ Gymnastik, Rytme, Spring, Tumbling<br>☐ Gåtur<br>☐ Håndbold, Basketball, Hockey<br>☐ Høvdingebold, Rundbold<br>☐ Is-skøjteløb, Ishockey<br>☐ Kano, kajak, roning<br>☐ Kampsport (f.eks. karate, boksning, taekwondo)<br>☐ Løb<br>☐ Parkour<br>☐ Ridning<br>☐ Rugby, Amerikansk fodbold<br>☐ Sejlsport<br>☐ Skateboard, Løbehjul, Rulleskøjter<br>☐ Styrketræning<br>☐ Svømning<br>☐ Volleyball<br>☐ Cyklet hjem fra skole<br>☐ Gået hjem fra skole<br>☐ Løbehjul, skateboard, rulleskøjter hjem fra skole<br>☐ Trampolin<br>☐ Andet
Hvor lang tid har du brugt på AEROBICS, DANS efter skole?	○ 0-15 minutter<br>○ 16-30 minutter<br>○ 31-45 minutter<br>○ 46-59 minutter<br>○ 1 time - 1 time og 15 minutter<br>○ 1 time og 16 minutter - 1 time og 30 minutter<br>○ 1time og 31 minutter - 1 time og 45 minutter<br>○ 1time og 46 minutter - 2 timer<br>○ Mere end 2 timer
Hvor lang tid har du brugt på ATLETIK efter skole?	○ 0-15 minutter<br>○ 16-30 minutter<br>○ 31-45 minutter<br>○ 46-59 minutter<br>○ 1 time - 1 time og 15 minutter<br>○ 1 time og 16 minutter - 1 time og 30 minutter<br>○ 1time og 31 minutter - 1 time og 45 minutter<br>○ 1time og 46 minutter - 2 timer<br>○ Mere end 2 timer

Hvor lang tid har du brugt på BADMINTON, TENNIS, SQUASH, BORDTENNIS efter skole?	◯ 0-15 minutter<br>◯ 16-30 minutter<br>◯ 31-45 minutter<br>◯ 46-59 minutter<br>◯ 1 time - 1 time og 15 minutter<br>◯ 1 time og 16 minutter - 1 time og 30 minutter<br>◯ 1time og 31 minutter - 1 time og 45 minutter<br>◯ 1time og 46 minutter - 2 timer<br>◯ Mere end 2 timer
Hvor lang tid har du brugt på BALLET efter skole?	◯ 0-15 minutter<br>◯ 16-30 minutter<br>◯ 31-45 minutter<br>◯ 46-59 minutter<br>◯ 1 time - 1 time og 15 minutter<br>◯ 1 time og 16 minutter - 1 time og 30 minutter<br>◯ 1time og 31 minutter - 1 time og 45 minutter<br>◯ 1time og 46 minutter - 2 timer<br>◯ Mere end 2 timer
Hvor lang tid har du brugt på BEACHVOLLEY efter skole?	◯ 0-15 minutter<br>◯ 16-30 minutter<br>◯ 31-45 minutter<br>◯ 46-59 minutter<br>◯ 1 time - 1 time og 15 minutter<br>◯ 1 time og 16 minutter - 1 time og 30 minutter<br>◯ 1time og 31 minutter - 1 time og 45 minutter<br>◯ 1time og 46 minutter - 2 timer<br>◯ Mere end 2 timer
Hvor lang tid har du brugt på CYKLING, SPINNING, KONDICYKEL efter skole?	◯ 0-15 minutter<br>◯ 16-30 minutter<br>◯ 31-45 minutter<br>◯ 46-59 minutter<br>◯ 1 time - 1 time og 15 minutter<br>◯ 1 time og 16 minutter - 1 time og 30 minutter<br>◯ 1time og 31 minutter - 1 time og 45 minutter<br>◯ 1time og 46 minutter - 2 timer<br>◯ Mere end 2 timer
Hvor lang tid har du brugt på E-SPORT efter skole?	◯ 0-15 minutter<br>◯ 16-30 minutter<br>◯ 31-45 minutter<br>◯ 46-59 minutter<br>◯ 1 time - 1 time og 15 minutter<br>◯ 1 time og 16 minutter - 1 time og 30 minutter<br>◯ 1time og 31 minutter - 1 time og 45 minutter<br>◯ 1time og 46 minutter - 2 timer<br>◯ Mere end 2 timer
Hvor lang tid har du brugt på FLOORBALL efter skole?	◯ 0-15 minutter<br>◯ 16-30 minutter<br>◯ 31-45 minutter<br>◯ 46-59 minutter<br>◯ 1 time - 1 time og 15 minutter<br>◯ 1 time og 16 minutter - 1 time og 30 minutter<br>◯ 1time og 31 minutter - 1 time og 45 minutter<br>◯ 1time og 46 minutter - 2 timer<br>◯ Mere end 2 timer

Hvor lang tid har du brugt på FODBOLD efter skole?	◯ 0-15 minutter<br>◯ 16-30 minutter<br>◯ 31-45 minutter<br>◯ 46-59 minutter<br>◯ 1 time - 1 time og 15 minutter<br>◯ 1 time og 16 minutter - 1 time og 30 minutter<br>◯ 1time og 31 minutter - 1 time og 45 minutter<br>◯ 1time og 46 minutter - 2 timer<br>◯ Mere end 2 timer
Hvor lang tid har du brugt på GEMMELEG, FIRKANT, SPARK TIL DÅSEN, FANGELEG/TIK efter skole?	◯ 0-15 minutter<br>◯ 16-30 minutter<br>◯ 31-45 minutter<br>◯ 46-59 minutter<br>◯ 1 time - 1 time og 15 minutter<br>◯ 1 time og 16 minutter - 1 time og 30 minutter<br>◯ 1time og 31 minutter - 1 time og 45 minutter<br>◯ 1time og 46 minutter - 2 timer<br>◯ Mere end 2 timer
Hvor lang tid har du brugt på GOLF efter skole?	◯ 0-15 minutter<br>◯ 16-30 minutter<br>◯ 31-45 minutter<br>◯ 46-59 minutter<br>◯ 1 time - 1 time og 15 minutter<br>◯ 1 time og 16 minutter - 1 time og 30 minutter<br>◯ 1time og 31 minutter - 1 time og 45 minutter<br>◯ 1time og 46 minutter - 2 timer<br>◯ Mere end 2 timer
Hvor lang tid har du brugt på GYMNASTIK efter skole?	◯ 0-15 minutter<br>◯ 16-30 minutter<br>◯ 31-45 minutter<br>◯ 46-59 minutter<br>◯ 1 time - 1 time og 15 minutter<br>◯ 1 time og 16 minutter - 1 time og 30 minutter<br>◯ 1time og 31 minutter - 1 time og 45 minutter<br>◯ 1time og 46 minutter - 2 timer<br>◯ Mere end 2 timer
Hvor lang tid har du brugt på GÅTUR efter skole?	◯ 0-15 minutter<br>◯ 16-30 minutter<br>◯ 31-45 minutter<br>◯ 46-59 minutter<br>◯ 1 time - 1 time og 15 minutter<br>◯ 1 time og 16 minutter - 1 time og 30 minutter<br>◯ 1time og 31 minutter - 1 time og 45 minutter<br>◯ 1time og 46 minutter - 2 timer<br>◯ Mere end 2 timer
Hvor lang tid har du brugt på HÅNDBOLD, BASKETBALL, HOCKEY efter skole?	◯ 0-15 minutter<br>◯ 16-30 minutter<br>◯ 31-45 minutter<br>◯ 46-59 minutter<br>◯ 1 time - 1 time og 15 minutter<br>◯ 1 time og 16 minutter - 1 time og 30 minutter<br>◯ 1time og 31 minutter - 1 time og 45 minutter<br>◯ 1time og 46 minutter - 2 timer<br>◯ Mere end 2 timer

Hvor lang tid har du brugt på HØVDINGEBOLD, RUNDBOLD efter skole?	◯ 0-15 minutter<br>◯ 16-30 minutter<br>◯ 31-45 minutter<br>◯ 46-59 minutter<br>◯ 1 time - 1 time og 15 minutter<br>◯ 1 time og 16 minutter - 1 time og 30 minutter<br>◯ 1time og 31 minutter - 1 time og 45 minutter<br>◯ 1time og 46 minutter - 2 timer<br>◯ Mere end 2 timer
Hvor lang tid har du brugt på IS-SKØJTELØB, ISHOCKEY efter skole?	◯ 0-15 minutter<br>◯ 16-30 minutter<br>◯ 31-45 minutter<br>◯ 46-59 minutter<br>◯ 1 time - 1 time og 15 minutter<br>◯ 1 time og 16 minutter - 1 time og 30 minutter<br>◯ 1time og 31 minutter - 1 time og 45 minutter<br>◯ 1time og 46 minutter - 2 timer<br>◯ Mere end 2 timer
Hvor lang tid har du brugt på KANO, KAJAK, RONING efter skole?	◯ 0-15 minutter<br>◯ 16-30 minutter<br>◯ 31-45 minutter<br>◯ 46-59 minutter<br>◯ 1 time - 1 time og 15 minutter<br>◯ 1 time og 16 minutter - 1 time og 30 minutter<br>◯ 1time og 31 minutter - 1 time og 45 minutter<br>◯ 1time og 46 minutter - 2 timer<br>◯ Mere end 2 timer
Hvor lang tid har du brugt på KAMPSPORT (f.eks. karate, boksning, teakwondo) efter skole?	◯ 0-15 minutter<br>◯ 16-30 minutter<br>◯ 31-45 minutter<br>◯ 46-59 minutter<br>◯ 1 time - 1 time og 15 minutter<br>◯ 1 time og 16 minutter - 1 time og 30 minutter<br>◯ 1time og 31 minutter - 1 time og 45 minutter<br>◯ 1time og 46 minutter - 2 timer<br>◯ Mere end 2 timer
Hvor lang tid har du brugt på LØB efter skole?	◯ 0-15 minutter<br>◯ 16-30 minutter<br>◯ 31-45 minutter<br>◯ 46-59 minutter<br>◯ 1 time - 1 time og 15 minutter<br>◯ 1 time og 16 minutter - 1 time og 30 minutter<br>◯ 1time og 31 minutter - 1 time og 45 minutter<br>◯ 1time og 46 minutter - 2 timer<br>◯ Mere end 2 timer
Hvor lang tid har du brugt på PARKOUR efter skole?	◯ 0-15 minutter<br>◯ 16-30 minutter<br>◯ 31-45 minutter<br>◯ 46-59 minutter<br>◯ 1 time - 1 time og 15 minutter<br>◯ 1 time og 16 minutter - 1 time og 30 minutter<br>◯ 1time og 31 minutter - 1 time og 45 minutter<br>◯ 1time og 46 minutter - 2 timer<br>◯ Mere end 2 timer

Hvor lang tid har du brugt på ridning efter skole?	◯ 0-15 minutter<br>◯ 16-30 minutter<br>◯ 31-45 minutter<br>◯ 46-59 minutter<br>◯ 1 time - 1 time og 15 minutter<br>◯ 1 time og 16 minutter - 1 time og 30 minutter<br>◯ 1time og 31 minutter - 1 time og 45 minutter<br>◯ 1time og 46 minutter - 2 timer<br>◯ Mere end 2 timer
Hvor lang tid har du brugt på Rugby, Amerikansk fodbold efter skole?	◯ 0-15 minutter<br>◯ 16-30 minutter<br>◯ 31-45 minutter<br>◯ 46-59 minutter<br>◯ 1 time - 1 time og 15 minutter<br>◯ 1 time og 16 minutter - 1 time og 30 minutter<br>◯ 1time og 31 minutter - 1 time og 45 minutter<br>◯ 1time og 46 minutter - 2 timer<br>◯ Mere end 2 timer
Hvor lang tid har du brugt på SEJLSPORT efter skole?	◯ 0-15 minutter<br>◯ 16-30 minutter<br>◯ 31-45 minutter<br>◯ 46-59 minutter<br>◯ 1 time - 1 time og 15 minutter<br>◯ 1 time og 16 minutter - 1 time og 30 minutter<br>◯ 1time og 31 minutter - 1 time og 45 minutter<br>◯ 1time og 46 minutter - 2 timer<br>◯ Mere end 2 timer
Hvor lang tid har du brugt på SKATEBOARD, LØBEHJUL, RULLESKØJTER efter skole?	◯ 0-15 minutter<br>◯ 16-30 minutter<br>◯ 31-45 minutter<br>◯ 46-59 minutter<br>◯ 1 time - 1 time og 15 minutter<br>◯ 1 time og 16 minutter - 1 time og 30 minutter<br>◯ 1time og 31 minutter - 1 time og 45 minutter<br>◯ 1time og 46 minutter - 2 timer<br>◯ Mere end 2 timer
Hvor lang tid har du brugt på STYRKETRÆNING efter skole?	◯ 0-15 minutter<br>◯ 16-30 minutter<br>◯ 31-45 minutter<br>◯ 46-59 minutter<br>◯ 1 time - 1 time og 15 minutter<br>◯ 1 time og 16 minutter - 1 time og 30 minutter<br>◯ 1time og 31 minutter - 1 time og 45 minutter<br>◯ 1time og 46 minutter - 2 timer<br>◯ Mere end 2 timer
Hvor lang tid har du brugt på SVØMNING efter skole?	◯ 0-15 minutter<br>◯ 16-30 minutter<br>◯ 31-45 minutter<br>◯ 46-59 minutter<br>◯ 1 time - 1 time og 15 minutter<br>◯ 1 time og 16 minutter - 1 time og 30 minutter<br>◯ 1time og 31 minutter - 1 time og 45 minutter<br>◯ 1time og 46 minutter - 2 timer<br>◯ Mere end 2 timer

Hvor lang tid har du brugt på VOLLEYBALL efter skole?	◯ 0-15 minutter<br>◯ 16-30 minutter<br>◯ 31-45 minutter<br>◯ 46-59 minutter<br>◯ 1 time - 1 time og 15 minutter<br>◯ 1 time og 16 minutter - 1 time og 30 minutter<br>◯ 1time og 31 minutter - 1 time og 45 minutter<br>◯ 1time og 46 minutter - 2 timer<br>◯ Mere end 2 timer
Hvor lang tid har du brugt på at GÅ hjem fra skole?	◯ 0-15 minutter<br>◯ 16-30 minutter<br>◯ 31-45 minutter<br>◯ 46-59 minutter<br>◯ 1 time - 1 time og 15 minutter<br>◯ 1 time og 16 minutter - 1 time og 30 minutter<br>◯ 1time og 31 minutter - 1 time og 45 minutter<br>◯ 1time og 46 minutter - 2 timer<br>◯ Mere end 2 timer
Hvor lang tid har du brugt på SKATEBOARD, LØBEHJUL, RULLESKØJTER hjem fra skole?	◯ 0-15 minutter<br>◯ 16-30 minutter<br>◯ 31-45 minutter<br>◯ 46-59 minutter<br>◯ 1 time - 1 time og 15 minutter<br>◯ 1 time og 16 minutter - 1 time og 30 minutter<br>◯ 1time og 31 minutter - 1 time og 45 minutter<br>◯ 1time og 46 minutter - 2 timer<br>◯ Mere end 2 timer
Hvor lang tid har du brugt på at CYKLE hjem fra skole?	◯ 0-15 minutter<br>◯ 16-30 minutter<br>◯ 31-45 minutter<br>◯ 46-59 minutter<br>◯ 1 time - 1 time og 15 minutter<br>◯ 1 time og 16 minutter - 1 time og 30 minutter<br>◯ 1time og 31 minutter - 1 time og 45 minutter<br>◯ 1time og 46 minutter - 2 timer<br>◯ Mere end 2 timer
Hvor lang tid har du brugt på at HOPPE TRAMPOLIN efter skole?	◯ 0-15 minutter<br>◯ 16-30 minutter<br>◯ 31-45 minutter<br>◯ 46-59 minutter<br>◯ 1 time - 1 time og 15 minutter<br>◯ 1 time og 16 minutter - 1 time og 30 minutter<br>◯ 1time og 31 minutter - 1 time og 45 minutter<br>◯ 1time og 46 minutter - 2 timer<br>◯ Mere end 2 timer
Hvilken anden aktivitet har du lavet efter skole?	◯ 0-15 minutter<br>◯ 16-30 minutter<br>◯ 31-45 minutter<br>◯ 46-59 minutter<br>◯ 1 time - 1 time og 15 minutter<br>◯ 1 time og 16 minutter - 1 time og 30 minutter<br>◯ 1time og 31 minutter - 1 time og 45 minutter<br>◯ 1time og 46 minutter - 2 timer<br>◯ Mere end 2 timer

Hvor lang tid har du brugt på denne aktivtet?	◯ 0-15 minutter<br>◯ 16-30 minutter<br>◯ 31-45 minutter<br>◯ 46-59 minutter<br>◯ 1 time - 1 time og 15 minutter<br>◯ 1 time og 16 minutter - 1 time og 30 minutter<br>◯ 1time og 31 minutter - 1 time og 45 minutter<br>◯ 1time og 46 minutter - 2 timer<br>◯ Mere end 2 timer
Hvilke fysiske aktiviteter har du lavet i aften, efter du har spist aftensmad?	☐ Ingen<br>☐ Aerobics, Dans<br>☐ Atletik<br>☐ Badminton, Tennis, Squash, Bordtennis<br>☐ Ballet<br>☐ Beachvolley<br>☐ Cykling, Spinning, Kondicykel<br>☐ E-sport<br>☐ Floorball<br>☐ Fodbold<br>☐ Gemmeleg, Firkant, Spark til dåsen, Fangeleg/Tik<br>☐ Golf<br>☐ Gymnastik, Rytme, Spring, Tumbling<br>☐ Gåtur<br>☐ Håndbold, Basketball, Hockey<br>☐ Høvdingebold, Rundbold<br>☐ Is-skøjteløb, Ishockey<br>☐ Kano, kajak, roning<br>☐ Kampsport (f.eks. karate, boksning, taekwondo)<br>☐ Løb<br>☐ Parkour<br>☐ Ridning<br>☐ Rugby, Amerikansk fodbold<br>☐ Sejlsport<br>☐ Skateboard, Løbehjul, Rulleskøjter<br>☐ Styrketræning<br>☐ Svømning<br>☐ Volleyball<br>☐ Trampolin<br>☐ Andet
Hvor lang tid har du brugt på AEROBICS, DANS i aften?	◯ 0-15 minutter<br>◯ 16-30 minutter<br>◯ 31-45 minutter<br>◯ 46-59 minutter<br>◯ 1 time - 1 time og 15 minutter<br>◯ 1 time og 16 minutter - 1 time og 30 minutter<br>◯ 1time og 31 minutter - 1 time og 45 minutter<br>◯ 1time og 46 minutter - 2 timer<br>◯ Mere end 2 timer
Hvor lang tid har du brugt på ATLETIK i aften?	◯ 0-15 minutter<br>◯ 16-30 minutter<br>◯ 31-45 minutter<br>◯ 46-59 minutter<br>◯ 1 time - 1 time og 15 minutter<br>◯ 1 time og 16 minutter - 1 time og 30 minutter<br>◯ 1time og 31 minutter - 1 time og 45 minutter<br>◯ 1time og 46 minutter - 2 timer<br>◯ Mere end 2 timer

Hvor lang tid har du brugt på BADMINTON, TENNIS, SQUASH, BORDTENNIS i aften?	◯ 0-15 minutter<br>◯ 16-30 minutter<br>◯ 31-45 minutter<br>◯ 46-59 minutter<br>◯ 1 time - 1 time og 15 minutter<br>◯ 1 time og 16 minutter - 1 time og 30 minutter<br>◯ 1time og 31 minutter - 1 time og 45 minutter<br>◯ 1time og 46 minutter - 2 timer<br>◯ Mere end 2 timer
Hvor lang tid har du brugt på BALLET i aften?	◯ 0-15 minutter<br>◯ 16-30 minutter<br>◯ 31-45 minutter<br>◯ 46-59 minutter<br>◯ 1 time - 1 time og 15 minutter<br>◯ 1 time og 16 minutter - 1 time og 30 minutter<br>◯ 1time og 31 minutter - 1 time og 45 minutter<br>◯ 1time og 46 minutter - 2 timer<br>◯ Mere end 2 timer
Hvor lang tid har du brugt på BEACHVOLLEY i aften?	◯ 0-15 minutter<br>◯ 16-30 minutter<br>◯ 31-45 minutter<br>◯ 46-59 minutter<br>◯ 1 time - 1 time og 15 minutter<br>◯ 1 time og 16 minutter - 1 time og 30 minutter<br>◯ 1time og 31 minutter - 1 time og 45 minutter<br>◯ 1time og 46 minutter - 2 timer<br>◯ Mere end 2 timer
Hvor lang tid har du brugt på CYKLING, SPINNING, KONDICYKEL i aften?	◯ 0-15 minutter<br>◯ 16-30 minutter<br>◯ 31-45 minutter<br>◯ 46-59 minutter<br>◯ 1 time - 1 time og 15 minutter<br>◯ 1 time og 16 minutter - 1 time og 30 minutter<br>◯ 1time og 31 minutter - 1 time og 45 minutter<br>◯ 1time og 46 minutter - 2 timer<br>◯ Mere end 2 timer
Hvor lang tid har du brugt på E-SPORT i aften?	◯ 0-15 minutter<br>◯ 16-30 minutter<br>◯ 31-45 minutter<br>◯ 46-59 minutter<br>◯ 1 time - 1 time og 15 minutter<br>◯ 1 time og 16 minutter - 1 time og 30 minutter<br>◯ 1time og 31 minutter - 1 time og 45 minutter<br>◯ 1time og 46 minutter - 2 timer<br>◯ Mere end 2 timer
Hvor lang tid har du brugt på FLOORBALL i aften?	◯ 0-15 minutter<br>◯ 16-30 minutter<br>◯ 31-45 minutter<br>◯ 46-59 minutter<br>◯ 1 time - 1 time og 15 minutter<br>◯ 1 time og 16 minutter - 1 time og 30 minutter<br>◯ 1time og 31 minutter - 1 time og 45 minutter<br>◯ 1time og 46 minutter - 2 timer<br>◯ Mere end 2 timer

Hvor lang tid har du brugt på FODBOLD i aften?	◯ 0-15 minutter<br>◯ 16-30 minutter<br>◯ 31-45 minutter<br>◯ 46-59 minutter<br>◯ 1 time - 1 time og 15 minutter<br>◯ 1 time og 16 minutter - 1 time og 30 minutter<br>◯ 1time og 31 minutter - 1 time og 45 minutter<br>◯ 1time og 46 minutter - 2 timer<br>◯ Mere end 2 timer
Hvor lang tid har du brugt på GEMMELEG, FIRKANT, SPARK TIL DÅSEN, FANGELEG/TIK, i aften?	◯ 0-15 minutter<br>◯ 16-30 minutter<br>◯ 31-45 minutter<br>◯ 46-59 minutter<br>◯ 1 time - 1 time og 15 minutter<br>◯ 1 time og 16 minutter - 1 time og 30 minutter<br>◯ 1time og 31 minutter - 1 time og 45 minutter<br>◯ 1time og 46 minutter - 2 timer<br>◯ Mere end 2 timer
Hvor lang tid har du brugt på GOLF i aften?	◯ 0-15 minutter<br>◯ 16-30 minutter<br>◯ 31-45 minutter<br>◯ 46-59 minutter<br>◯ 1 time - 1 time og 15 minutter<br>◯ 1 time og 16 minutter - 1 time og 30 minutter<br>◯ 1time og 31 minutter - 1 time og 45 minutter<br>◯ 1time og 46 minutter - 2 timer<br>◯ Mere end 2 timer
Hvor lang tid har du brugt på GYMNASTIK i aften?	◯ 0-15 minutter<br>◯ 16-30 minutter<br>◯ 31-45 minutter<br>◯ 46-59 minutter<br>◯ 1 time - 1 time og 15 minutter<br>◯ 1 time og 16 minutter - 1 time og 30 minutter<br>◯ 1time og 31 minutter - 1 time og 45 minutter<br>◯ 1time og 46 minutter - 2 timer<br>◯ Mere end 2 timer
Hvor lang tid har du brugt på GÅTUR i aften?	◯ 0-15 minutter<br>◯ 16-30 minutter<br>◯ 31-45 minutter<br>◯ 46-59 minutter<br>◯ 1 time - 1 time og 15 minutter<br>◯ 1 time og 16 minutter - 1 time og 30 minutter<br>◯ 1time og 31 minutter - 1 time og 45 minutter<br>◯ 1time og 46 minutter - 2 timer<br>◯ Mere end 2 timer
Hvor lang tid har du brugt på HÅNDBOLD, BASKETBALL, HOCKEY i aften?	◯ 0-15 minutter<br>◯ 16-30 minutter<br>◯ 31-45 minutter<br>◯ 46-59 minutter<br>◯ 1 time - 1 time og 15 minutter<br>◯ 1 time og 16 minutter - 1 time og 30 minutter<br>◯ 1time og 31 minutter - 1 time og 45 minutter<br>◯ 1time og 46 minutter - 2 timer<br>◯ Mere end 2 timer

Hvor lang tid har du brugt på HØVDINGEBOLD, RUNDBOLD i aften?	◯ 0-15 minutter<br>◯ 16-30 minutter<br>◯ 31-45 minutter<br>◯ 46-59 minutter<br>◯ 1 time - 1 time og 15 minutter<br>◯ 1 time og 16 minutter - 1 time og 30 minutter<br>◯ 1time og 31 minutter - 1 time og 45 minutter<br>◯ 1time og 46 minutter - 2 timer<br>◯ Mere end 2 timer
Hvor lang tid har du brugt på IS-SKØJTELØB, ISHOCKEY i aften?	◯ 0-15 minutter<br>◯ 16-30 minutter<br>◯ 31-45 minutter<br>◯ 46-59 minutter<br>◯ 1 time - 1 time og 15 minutter<br>◯ 1 time og 16 minutter - 1 time og 30 minutter<br>◯ 1time og 31 minutter - 1 time og 45 minutter<br>◯ 1time og 46 minutter - 2 timer<br>◯ Mere end 2 timer
Hvor lang tid har du brugt på KANO, KAJAK. RONING I AFTEN?	◯ 0-15 minutter<br>◯ 16-30 minutter<br>◯ 31-45 minutter<br>◯ 46-59 minutter<br>◯ 1 time - 1 time og 15 minutter<br>◯ 1 time og 16 minutter - 1 time og 30 minutter<br>◯ 1time og 31 minutter - 1 time og 45 minutter<br>◯ 1time og 46 minutter - 2 timer<br>◯ Mere end 2 timer
Hvor lang tid har du brugt på KAMPSPORT (f.eks. karate, boksning, teakwondo) i aften?	◯ 0-15 minutter<br>◯ 16-30 minutter<br>◯ 31-45 minutter<br>◯ 46-59 minutter<br>◯ 1 time - 1 time og 15 minutter<br>◯ 1 time og 16 minutter - 1 time og 30 minutter<br>◯ 1time og 31 minutter - 1 time og 45 minutter<br>◯ 1time og 46 minutter - 2 timer<br>◯ Mere end 2 timer
Hvor lang tid har du brugt på LØB i aften?	◯ 0-15 minutter<br>◯ 16-30 minutter<br>◯ 31-45 minutter<br>◯ 46-59 minutter<br>◯ 1 time - 1 time og 15 minutter<br>◯ 1 time og 16 minutter - 1 time og 30 minutter<br>◯ 1time og 31 minutter - 1 time og 45 minutter<br>◯ 1time og 46 minutter - 2 timer<br>◯ Mere end 2 timer
Hvor lang tid har du brugt på PARKOUR i aften?	◯ 0-15 minutter<br>◯ 16-30 minutter<br>◯ 31-45 minutter<br>◯ 46-59 minutter<br>◯ 1 time - 1 time og 15 minutter<br>◯ 1 time og 16 minutter - 1 time og 30 minutter<br>◯ 1time og 31 minutter - 1 time og 45 minutter<br>◯ 1time og 46 minutter - 2 timer<br>◯ Mere end 2 timer

Hvor lang tid har du brugt på SEJLSPORT i aften?

- ◯ 0-15 minutter
- ◯ 16-30 minutter
- ◯ 31-45 minutter
- ◯ 46-59 minutter
- ◯ 1 time - 1 time og 15 minutter
- ◯ 1 time og 16 minutter - 1 time og 30 minutter
- ◯ 1time og 31 minutter - 1 time og 45 minutter
- ◯ 1time og 46 minutter - 2 timer
- ◯ Mere end 2 timer

Hvor lang tid har du brugt på AT RIDE i aften?

- ◯ 0-15 minutter
- ◯ 16-30 minutter
- ◯ 31-45 minutter
- ◯ 46-59 minutter
- ◯ 1 time - 1 time og 15 minutter
- ◯ 1 time og 16 minutter - 1 time og 30 minutter
- ◯ 1time og 31 minutter - 1 time og 45 minutter
- ◯ 1time og 46 minutter - 2 timer
- ◯ Mere end 2 timer

Hvor lang tid har du brugt på Rugby, Amerikansk fodbold i aften?

- ◯ 0-15 minutter
- ◯ 16-30 minutter
- ◯ 31-45 minutter
- ◯ 46-59 minutter
- ◯ 1 time - 1 time og 15 minutter
- ◯ 1 time og 16 minutter - 1 time og 30 minutter
- ◯ 1time og 31 minutter - 1 time og 45 minutter
- ◯ 1time og 46 minutter - 2 timer
- ◯ Mere end 2 timer

Hvor lang tid har du brugt på SKATEBOARD, LØBEHJUL, RULLESKØJTER i aften?

- ◯ 0-15 minutter
- ◯ 16-30 minutter
- ◯ 31-45 minutter
- ◯ 46-59 minutter
- ◯ 1 time - 1 time og 15 minutter
- ◯ 1 time og 16 minutter - 1 time og 30 minutter
- ◯ 1time og 31 minutter - 1 time og 45 minutter
- ◯ 1time og 46 minutter - 2 timer
- ◯ Mere end 2 timer

Hvor lang tid har du brugt på STYRKETRÆNING i aften?

- ◯ 0-15 minutter
- ◯ 16-30 minutter
- ◯ 31-45 minutter
- ◯ 46-59 minutter
- ◯ 1 time - 1 time og 15 minutter
- ◯ 1 time og 16 minutter - 1 time og 30 minutter
- ◯ 1time og 31 minutter - 1 time og 45 minutter
- ◯ 1time og 46 minutter - 2 timer
- ◯ Mere end 2 timer

Hvor lang tid har du brugt på SVØMNING i aften?

- ◯ 0-15 minutter
- ◯ 16-30 minutter
- ◯ 31-45 minutter
- ◯ 46-59 minutter
- ◯ 1 time - 1 time og 15 minutter
- ◯ 1 time og 16 minutter - 1 time og 30 minutter
- ◯ 1time og 31 minutter - 1 time og 45 minutter
- ◯ 1time og 46 minutter - 2 timer
- ◯ Mere end 2 timer

Hvor lang tid har du brugt på VOLLEYBALL i aften?	◯ 0-15 minutter<br>◯ 16-30 minutter<br>◯ 31-45 minutter<br>◯ 46-59 minutter<br>◯ 1 time - 1 time og 15 minutter<br>◯ 1 time og 16 minutter - 1 time og 30 minutter<br>◯ 1time og 31 minutter - 1 time og 45 minutter<br>◯ 1time og 46 minutter - 2 timer<br>◯ Mere end 2 timer
Hvor lang tid har du brugt på at HOPPE TRAMPOLIN i aften?	◯ 0-15 minutter<br>◯ 16-30 minutter<br>◯ 31-45 minutter<br>◯ 46-59 minutter<br>◯ 1 time - 1 time og 15 minutter<br>◯ 1 time og 16 minutter - 1 time og 30 minutter<br>◯ 1time og 31 minutter - 1 time og 45 minutter<br>◯ 1time og 46 minutter - 2 timer<br>◯ Mere end 2 timer
Hvilken anden fysisk aktivitet har du lavet i aften?	______________________
Hvor lang tid har du brugt på denne aktivitet?	◯ 0-15 minutter<br>◯ 16-30 minutter<br>◯ 31-45 minutter<br>◯ 46-59 minutter<br>◯ 1 time - 1 time og 15 minutter<br>◯ 1 time og 16 minutter - 1 time og 30 minutter<br>◯ 1time og 31 minutter - 1 time og 45 minutter<br>◯ 1time og 46 minutter - 2 timer<br>◯ Mere end 2 timer

MyCap App Fields - Do Not Modify

UUID	______________________
Start Date	______________________
End Date	______________________
Schedule Date	______________________
Status	◯ Deleted<br>◯ Completed<br>◯ Incomplete
Supplemental Data (JSON)	___________________________
Serailized Result	
