# Supplementary material for: Adaptation of a Danish online version of the Oxford Physical Activity Questionnaire (OPAQ) for secondary school students—a pilot study
Source: Pilot Feasibility Stud. 2022 Jul 25;8:153. doi: 10.1186/s40814-022-01108-x (PMC9309605; doi:10.1186/s40814-022-01108-x)
Supplement: Supplementary file 4 — Additional file 4. Linguistic translation and cross-cultural adaptation. [file 40814_2022_1108_MOESM4_ESM.pdf]

Appendix 4

Linguistic translation and cross-cultural adaptation

No major issues were encountered in the translation of OPAQ from English to Danish. Only minor discrepancies between the three Danish translations were observed and resolved by consensus. For example, "tick" was translated in two different ways (mark, cross) as "tick" can´t be translated with one word in Danish. The question "cultural background" and "school sport" were subjects for discussion because of the cultural discrepancies between Denmark and Australia. The primary issue about "cultural background" was how to replace Australian, Aboriginal, Torres Strait Islander, Asian, European, Middle Eastern, African, Other (please specify). Therefore, we chose to have only Danish and other (please specify).

In the Danish public-school system, we do not have sports teams and tournaments wherein schools compete against each other. Therefore, we removed "school sport" from the questionnaire.

Furthermore, all translators agreed on adding and removing some of the suggested activities in the scheme based on cultural adaptations so that the list of activities were comprehensible and suitable for use in Denmark. For example, handball, badminton, and horse-riding are very common sports in Denmark, but touch football, netball, lifesaving, and cricket are not.

The synthesis of the backtranslation into English by two independent translators with English as their first language was approved by the authors of the original version of OPAQ. No issues were noted in the approval by the original authors.

During the interviews of the 12 school students aged 10-15 (five girls and seven boys, two in each year-group) they reported that the Danish version of OPAQ was easy to understand in general, and that the questions were relevant for tracking their activities during the week.

The interviews revealed a few difficulties concerning recalling activities and understanding "what is your cultural background" by five of the participants. Therefore, the expert committee agreed to replace this question with two questions which is more suitable for use in Denmark; "Country of birth?" and "Which languages do you primary speak at home".

The students who received the questionnaire in the end of the week experienced it hard to recall seven days. They spent 15 minutes on filling in the questionnaire. The students who received the questionnaire in the beginning of the week and replied every evening had no troubles recalling their activities during the day. They used maximum three minutes per day.

Most (9/12) of the students did not read the explanation on how to fill in the timetable in the questionnaire and which activities to note. This was reflected in their response. Two were unsure how to fill in and two were unsure which PA to note.

Ten did not note every PA they had done during the week. For example, they did not think of transportation by bike to sport as PA, and two of the twelve adolescents forgot to note PE. They suggested that showing only one box at a time combined with a hint like: “Note if you have done any sport today. Remember to note if your transportation was active as well” or “have you done PE or other PA during the school lessons today”, would be very useful memories cues. In addition, they suggested that parents could remind them fill in the questionnaire every day.
